# Characterization of *pncA* Mutations and Prediction of PZA Resistance in *Mycobacterium tuberculosis* Clinical Isolates From Chongqing, China

**DOI:** 10.3389/fmicb.2020.594171

**Published:** 2021-01-11

**Authors:** Kun Li, Zhongping Yang, Jing Gu, Ming Luo, Jiaoyu Deng, Yaokai Chen

**Affiliations:** ^1^State Key Laboratory of Silkworm Genome Biology, Southwest University, Chongqing, China; ^2^Central Laboratory, Chongqing Public Health Medical Center, Chongqing, China; ^3^Key Laboratory of Special Pathogens and Biosafety, Wuhan Institute of Virology, Chinese Academy of Sciences, Wuhan, China

**Keywords:** tuberculosis, pyrazinamide, *pncA* mutations, PZase activity, *Escherichia coli*

## Abstract

Pyrazinamide (PZA) is widely used to treat drug-sensitive or multidrug resistance tuberculosis. However, conventional PZA susceptibility tests of clinical isolates are rather difficult because of the requirement of acid pH. Since resistance to pyrazinamide is primary mediated by mutation of *pncA*, an alternative way of PZA susceptibility test is to analyze the pyrazinamidase activities of *Mycobacterium tuberculosis* clinical isolates. Therefore, a database containing the full spectrum of *pncA* mutations along with pyrazinamidase activities will be beneficial. To characterize mutations of *pncA* in *M. tuberculosis* from Chongqing, China, the *pncA* gene was sequenced and analyzed in 465 clinical isolates. A total of 124 types of mutations were identified in 424 drug-resistant isolates, while no mutation was identified in the 31 pan-susceptible isolates. Ninety-four of the 124 mutations had previously been reported, and 30 new mutations were identified. Based on reported literatures, 294 isolates could be predicted resistant to pyrazinamide. Furthermore, pyrazinamidase activities of the 30 new mutations were tested using the *Escherichia coli pncA* gene knockout strain. The results showed that 24 of these new mutations (28 isolates) led to loss of pyrazinamidase activity and six (8 isolates) of them did not. Taken together, 322 isolates with *pncA* mutations could be predicted to be PZA resistant among the 424 drug-resistant isolates tested. Analysis of *pncA* mutations and their effects on pyrazinamidase activity will not only enrich our knowledge of comprehensive *pncA* mutations related with PZA resistance but also facilitate rapid molecular diagnosis of pyrazinamide resistance in *M. tuberculosis*.

## Introduction

Nowadays, *Mycobacterium tuberculosis* (*M. tuberculosis*) is still one of the most important human pathogens worldwide, which caused about 1.45 million deaths in 2018 (World Health Organization [WHO], 2019). The emergence of multidrug (MDR) and extensively resistant (XDR) *M. tuberculosis* strains makes it even more difficult to control the disease of tuberculosis (TB). Pyrazinamide (PZA), a synthetic derivative of nicotinamide, was firstly synthesized in 1936, and subsequently used for the treatment of TB since 1952 (Palomino and Martin, 2014). Till present, PZA has been listed as a first-line agent in the modern short-course TB regimen. Due to its unique bactericidal activity against slow growing or metabolically inactive *M. tuberculosis* that is not killed by other TB drugs, it shortens the time of therapy from 9 to 12 to 6 months and is used in treatment of both drug-susceptible *M. tuberculosis* and PZA-susceptible MDR *M. tuberculosis* (Mitchison, 1985; Zhang and Mitchison, 2003).

Although having been used in the treatment of *M. tuberculosis* for more than 60 years, the accurate mode of action of PZA still remains elusive. The most widely accepted mechanism is that PZA is a prodrug and needs to be converted to its active form of pyrazinoic acid (POA) by the pyrazinamidase (PZase) encoded by the *pncA* gene. POA as an ionophore in the cytosol is expelled by a weak efflux pump into the acidic conditions. The protonated POA re-enters into cytosol and releases proton. Because of the inefficiency of the efflux pump, repetition of this cycle causes POA accumulation and acidification in cytosol and leads to cellular damage (Zhang et al., 1999). Mutation of the *pncA* gene aborts or reduces PZase activity and is the major reason why clinical *M. tuberculosis* strains acquire PZA resistance. However, some clinical PZA-resistant strains with positive PZase do not have *pncA* gene mutations, suggesting the existence of alternative mechanisms (Zhang and Mitchison, 2003).

So far, the real target of POA has never been identified. Overexpression of fatty acid synthetase I (FAS-I) encoded by eukaryotic-like *fas1* gene from *M. tuberculosis*, *M. bovis BCG*, or *Mycobacterium avium* in *Mycobacterium smegmatis* confers resistance to 5-Cl-PZA, a PZA analog, and FAS-I used to be thought as the target of PZA (Zimhony et al., 2000). However, it was lately realized that POA failed to directly inhibits mycobacterial fatty acid synthase FAS-I, and this enzyme is the target of 5-Cl-PZA, not PZA (Boshoff et al., 2002). The ribosomal protein S1, a vital protein involved in ribosome-sparing process of trans-translation, was also speculated to be the target of POA. Overexpression of RpsA did confer PZA resistance, and three clinical PZA-resistant isolates with no mutation in *pncA*-harbored RpsA mutations (Shi et al., 2011). However, later research showed that *M. tuberculosis* RpsA interacts with single-stranded RNA, but not with POA (Dillon et al., 2017), and strains with deletion of trans-translation-relevant genes did not show any change of PZA susceptibility (Personne and Parish, 2014). The aspartate decarboxylase encoded by *panD* is involved in synthesis of β-alanine, which is a precursor for the biosynthesis of co-enzyme A and pantothenate. Whole genome sequencing analyses demonstrated that PZA-resistant clinical isolates lacking *pncA* and *rpsA* mutations were observed with *panD* mutations (Zhang et al., 2013). On the other hand, although supplementation of precursors of coenzyme A biosynthesis, such as β-alanine, pantothenate, or pantetheine could antagonize the action of PZA, the gene deletion of pantothenate biosynthesis pathway did not antagonize PZA activity. This finding demonstrated that pantothenate biosynthesis pathway including *panD* is not involved in PZA activity (Dillon et al., 2014). Very recently, researchers found that an ATP-dependent ATPase (ClpC1) involved in protein degradation (Zhang et al., 2017), and a bifunctional enzyme Rv2783 involved in metabolism of RNA (Njire et al., 2017) also confer PZA resistance in clinical isolates. Though evidences have shown that mutations of *rpsA*, *panD*, and *clpC1* are involved in PZA resistance, contributions of those mutations to clinical resistance of PZA in *M. tuberculosis* still need further investigation.

Though drug susceptibility testing (DST) is recommended for both first- and second-line anti-tuberculosis drugs (World Health Organization [WHO], 2019), DST for PZA is complicated due to the requirement of acidic pH (Zhang et al., 2002). At present, Bactec MGIT 960 system (Becton Dickinson Biosciences, Sparks, MD) still has unreliable results presented (Chedore et al., 2010; Piersimoni et al., 2013). In some cases, molecular test is rapid, simple, and reliable for prediction of drug resistance. Although the exact mechanism of PZA action is still in research, a couple of genes involved in PZA resistance have been found as mentioned above. One of most important gene is *pncA*, which accounts for 70–97% of the PZA resistance mutations (Hirano et al., 1997). However, unlike the resistance conferring regions of *rpoB* to rifampicin, *katG* to isoniazid, no clear hotspot for *pncA* mutations has been found. Mutations are highly diverse and scattered throughout the *pncA* gene, complicating the molecular assessment of PZA resistance and making DNA sequencing the best option so far (Miotto et al., 2014). Moreover, there are reports showing that some specific *pncA* mutations observed in clinical isolates do not affect the susceptibility to PZA (Xia et al., 2015; Allix-Béguec et al., 2018). As an assistant PZA susceptibility judgment method, classical PZase tests, namely Wayne’s method, used orange-red color change of POA to react with ammonium ferrous sulfate (Wayne, 1974). Subsequent modified Wayne’s method (Zhang et al., 2013; Aono et al., 2018) omitted protein precipitate step which used trichloroacetic acid. However, all the reported Wayne’s methods need large amount of *M. tuberculosis* culture and hence a long growing cycle is inevitable. In addition, so far, all methods based on Wayne’s work need to be performed in high level biosafety labs (BSL-3) due to the requirement of large amount of bacterial cultures.

Since over 60% MDR TB cases globally are PZA resistant (Whitfield et al., 2015), and the vital role of PZA in the treatment of TB, rapid and easy to perform molecular assays for PZA susceptibility tests are highly desirable. To achieve this, the *pncA* genes of 465 clinical *M. tuberculosis* isolates from Chongqing district were sequenced and mutations were analyzed. Subsequently, PZA susceptibilities of the isolates containing previous reported *pncA* mutations were predicted based on reported literatures. In addition, an *Escherichia coli pncA* gene knockout strain was used to analyze the PZase activities of the newly identified *pncA* mutations and hence predict the PZA susceptibilities of the corresponding isolates. The results were reported herein.

## Materials and Methods

### Clinical *M. tuberculosis* Isolates

A total of 465 randomly selected clinical samples from different patients (Table 1) mostly came from Chongqing local and surrounding areas between April 2014 and August 2018 in Chongqing Public Health Medical Center (Chongqing, China) were cultured in BACTEC MGIT 960 system (Becton Dickinson, Sparks, MD, United States), according to manufacturer’s instructions (Kruuner et al., 2006). The flagged positive samples were subjected to DST using proportion method on Lowenstein-Jensen (L-J) solid medium (Encode, Zhuhai, China) containing MDR- and XDR-related drugs, isoniazid, rifampicin, ofloxacin, levofloxacin, moxifloxacin, amikacin, and capreomycin. The *Mycobacterium* species identification were assigned based on sequence polymorphisms in 16S *rRNA*, *hsp65*, and *rpoB* (Kim and Shin, 2018), and on the results of 5 μg/ml TCH and 500 μg/ml PNB on L-J solid medium. Isolates were frozen in 25% glycerol at −70°C refrigerator until use.

**TABLE 1:** Classification of 465 isolates used in this study.

**Sample type**	***n***	**%**	**Age**	***n***	**%**	**Drug susceptibility profile**	***n***	**%**
Sputum	351	75.5	≤24	90	19.4	Pan-susceptible	31	6.7
Fiberoptic bronchoscopy lavage fluid	53	11.4	25–44	196	42.2	Mono	19	4.1
Pleural fluid	10	2.2	45–64	152	32.7	MDR	310	66.7
Cerebrospinal fluid	5	1.1	≥65	27	5.8	XDR	76	16.3
Ascitic fluid	1	0.2	Gender	*n*	%	Other^*b*^	29	6.2
Pyogenic fluid	24	5.2	Female	157	33.8			
Other^*a*^	21	4.5	Male	308	66.2			

**Other^*a*^, samples not belonging to above six types; Pan-susceptible, sensitive to MDR/XDR-related drugs; Mono, resistant to one of MDR/XDR-related drugs; Other^*b*^, resistant to drugs not related to Mono, MDR, or XDR.**

### Mycobacterial Genomic DNA

Colonies of isolates on L-J solid medium were transferred with several loopfuls into 1.5 ml Eppendorf tubes and suspended in 1 ml deionized water, and then centrifuged for DNA extraction. DNA isolation was conducted using HiPure Mycobacterial DNA Kit (Magen, Guangzhou, China). In brief, the mycobacterial cell pellet was washed twice with deionized water and suspended with 200 μl GTL containing lysozyme (10 μg/ml) and proteinase K (10 μg/ml). The bacterial cells were incubated at 37°C for 20 min, 56°C for 20 min, 95°C for 20 min and then mixed with 600 μl GXP. After centrifugation, the DNA in supernatant was collected on silica membrane, washed with GDW, and resuspended in TE buffer.

### *pncA* Gene Amplification and Sequencing Analysis

The entire open reading frame of *pncA* flanking 150 bp of upstream putative regulatory sequence was amplified using forward primer *pncA*-F (5′-GGCCCGATGAAGGTGTCGTA) and reverse primer *pncA*-R (5′-CGGACGGATTTGTCGCTCA CTAC). The *pncA* primers were designed according to the *M. tuberculosis* reference sequence (GenBank accession number AL123456.3).

A S1000 Thermal Cycler (BIO-RAD, Hercules, CA, United States) was used for amplification of *pncA* with the following procedures: a predegeneration step at 98°C for 10 min, degeneration at 98°C for 15 s, annealing at 61.2°C for 15 s, extension at 72°C for 10 s, and terminated with final extension at 72°C for 5 min. Agarose gel electrophoresis was performed, and the DNA was purified with a Omega Kit (Omega Bio-tek, Norcross, GA, United States) according to the manufacturer’s instructions. The purified DNA products were sequenced in a DNA sequencer (ABI, model 3730XL, Carlsbad, CA, United States) with primers same as the PCR amplification. The sequencing results were compared with the reference sequence using nucleotide BLAST^1^, and the mutation polymorphisms were depicted according to Sequence Variant Nomenclature^2^.

### PZase Activity Tests in *Escherichia coli* W3110Δ*pncA*

Based on Wayne’s method (Wayne, 1974; Zhang et al., 2013; Aono et al., 2018), a modified protocol was used to test the PZase activities of the 30 new mutations identified in this study, by using fast-growing *E. coli*. Plasmids pKD4, pKD46, pCP20, pCA24N, and *E. coli* K12 strain W3110 were kindly provided by TB research group in Wuhan Institute of Virology of Chinese Academy of Sciences. *E. coli* W3110 Δ *pncA* strain was constructed according to a previous publication (Li et al., 2017). Primers used for construction (*pncA*-ko-s, *pncA*-ko-a), verification (*pncA*-jd-s, *pncA*-jd-a) of *E. coli* W3110 Δ *pncA*, and *pncA* amplification (*pncA*-f, *pncA*-r) from clinical TB strains are listed in Table 2. All the culture mediums and antibiotics were purchased from Merck (Darmstadt, Germany) and restriction endonucleases from TAKARA (Tokyo, Japan).

**TABLE 2:** Primers used in construction of *E. coli* W3110 Δ *pncA* strain and amplification of *M. tuberculosis pncA*.

**Primers**	Sequences*
*pncA*-ko-s	5-atgccccctcgcgccctgttactggtcgatttacaaaatgatttctgtgc
	atatgaatatcctccttag-3
*pncA*-ko-a	5-ttacccctgtgtctcttcccagtctgccagcgtatatagcgttgccccag
	tgtaggctggagctgcttcg-3
*pncA*-jd-s	5-cggtaaagtgaacatgggtg-3
*pncA*-jd-a	5-gcctacaatgccagtacagaa-3
*pncA*-f	5-cctgagggccagatctatgcgggcgttgatcatcgtc-3
*pncA*-r	5-tagacccggggagctctcaggagctgcaaaccaac-3

*Sequences underlined were matched to pncA in the genome of E. coli K12 W3110.

For PZase activity tests, products of *pncA* in clinical TB strains were amplified, purified, digested using *Sac*II and *Bgl*II, and then ligated to the same digested plasmid pCA24N. The verified pCA24N-*pncA* was transformed into *E. coli* W3110 Δ *pncA*. Recombinant strains transformed with *pncA* genes from different *M. tuberculosis* clinical isolates were cultured in 37°C incubator to mid-log phase (OD_600_, 0.7∼1.0), then 10 ml culture of each strain was used and a final concentration of 100 μg/ml pyrazinamide was added. After overnight incubation, final concentration of 2% ammonium ferrous sulfate was added into 2 ml culture supernatant of each strain and optical density at wave length 530 nm (OD_530_) was measured. The strain contained wild-type *pncA* from *M. tuberculosis* H37Rv was set as positive control and with the vector as negative control. Cutoff value was set as the ratio between the sample and the negative control that is more than 2.1 (Yamashita et al., 1995).

### Drug Susceptibility Testing of PZA

Isolates stored at −70°C refrigerators were quickly thawed in 37°C water bath and then streaked on 7H10 solid medium (Difco, Becton, Dickinson and company, Sparks, MD, United States) supplemented with 10% (*V*/*V*) oleic-albumin-dextrose-catalase (OADC, Difco) and 0.5% (*V*/*V*) glycerin. After 3–4 weeks of incubation under 37°C, colonies on 7H10 plates were picked into 7H9 liquid medium (Difco) for another 3–4 weeks of incubation. The 7H10 medium used for PZA DST were adjusted to pH 5.7–5.8 using hydrochloric acid and then steam sterilized at temperature of 121°C for 15 min, afterward OADC and glycerin were added when the medium were cooled to 50°C. PZA (Merck, Germany) powder was dissolved in dimethyl sulfoxide (Merck, Germany) to final concentration of 60 mg/ml. The 7H10 plates containing serial concentrations of PZA (0, 6.25, 12.5, 25, 50, 100, 200, and 400 μg/ml) were prepared. Bacteria cells in mid-log phase cultured in 7H9 medium were adjusted to McFarland concentration of 1.0 and then diluted to 100-fold using physiological saline solution. Aliquot of 10 μl diluents were dropped onto the 7H10 medium with serial concentrations of PZA and incubated at 37°C for 4 weeks. *M. tuberculosis* H37Rv (ATCC 27294) was set as the control. Minimum inhibitory concentration (MIC) is defined as the lowest drug concentration that inhibits 99% growth of the bacteria.

## Results

### Sample Characteristics

Characteristics of all the selected *M. tuberculosis* strains are listed in Table 1. The sputum samples accounted for the largest proportion (75.5%). The age bracket 25–44 made up 42.2% in the total samples, illustrating TB are mainly popular in young adults. The male patients (66.2%) were nearly two times more than the female patients (33.8%). According to the DST results of MDR- and XDR-relevant drugs, in the 465 clinical isolates, there are 310 (66.7%) MDR and 76 (16.3%) XDR TB isolates.

### Mutations in the *pncA* Gene

The *pncA* gene of 465 clinical isolates was sequenced. Of these, 346 (74.4%) isolates harbored at least one mutation compared with the wild-type reference sequence (Table 3). For the 31 isolates pan susceptible to MDR- and XDR-related drugs, no mutations in the *pncA* coding zone and putative upstream regulatory region were identified. Among the 19 mono (resistant to one of the MDR/XDR related drugs), 29 Other (resistant to drugs not related to MDR/XDR), 310 MDR and 76 XDR TB isolates, 2 (10.5%), 16 (55.2%), 254 (81.9%), and 74 (97.4%) of them harbored mutations in the *pncA* gene (including its upstream region), respectively (Table 3). Of the 124 types of mutations identified, three mutations at positions −12 and −11 upstream of *pncA* were identified in 11 isolates (Supplementary Table S1). Except for those three mutations, the remaining 121 mutations were distributed within the *pncA* open reading frame. Among the mutations observed in this study, 94 have previously been reported, 30 have not (Supplementary Tables S1, S4). We only found one synonymous mutation in the 124 mutational types and the rest of them were non-synonymous mutations (123 of 124). The majority of these mutations were nucleotide substitution (100 of 124) which caused amino acid substitution, 18 types with insertions, and six types with deletions (Supplementary Table S2). Seven types of mutations causing protein translation stop at amino acid positions 10, 41, 68, 103, 119, 122, and 141 were identified in 11 isolates (Supplementary Tables S1, S4).

**TABLE 3:** *pncA* mutation profiles in clinical TB isolates resistant to MDR- and XDR-relevant drugs.

**Resistance profile**	**No. (%) of mutations**	**Wild type**
Pan-susceptible (*n* = 31)	0	31
Mono (*n* = 19)	2 (10.5%)	17
Other^*b*^ (*n* = 29)	16 (55.2%)	13
MDR-TB (*n* = 310)	254 (81.9%)	56
XDR-TB (*n* = 76)	74 (97.4%)	2
Total (*n* = 465)	346 (74.4%)	119

**Other^*b*^, strains resistant to drugs not related to Mono, MDR or XDR.**

The most frequent change observed in this study was 407_408 *insA*, which was found in 51 isolates (Supplementary Tables S1, S4). Meanwhile, 33 isolates contained a point mutation at position 395 and 40 isolates had mutation at position 226. We also observed four isolates had double mutations, 193T > C with 359T > C (1 isolate), 176C > A with 254T > C (1 isolate), 257A > G with 538G > T (1 isolate), and 310A > G with 388G > A (1 isolate). No *M. bovis* BCG-specific mutation 169C > G was observed in this study. The detailed information of all mutations is listed in Supplementary Table S1.

### Correlation of Previously Reported *pncA* Mutations to PZA Susceptibility and PZase Activity

Due to technical difficulties, PZA MIC tests are not available in most of the clinical hospitals. Therefore, the MICs and PZase activities of isolates with previously reported mutations were collected and are listed in Supplementary Table S4 based on previous publications. Among the 465 samples, 310 contained mutations previously reported. Resistance profile to PZA and PZase activities (negative or positive) were summarized in Table 4. 294 isolates were predicted to be resistant to PZA and 273 were predicted to be PZase negative. There were 211 (68.1%) and 67 (88.2%) isolates predicted to be resistant to PZA in MDR and XDR strains, respectively. Among the 94 types of mutations previously reported, T > G at position 40 and C > T at position 176 were consistently reported susceptible to PZA with the MIC < 100 μg/ml (Stoffels et al., 2012; Xia et al., 2015); A > G at position 35 and 139 and A > C at positions 143 and 403 had literatures showing inconsistent MIC results at a PZA concentration of 100 μg/ml (Morlock et al., 2000; Somoskovi et al., 2007; Juréen et al., 2008; Mphahlele et al., 2008; Stoffels et al., 2012; Xia et al., 2015; Ramirez-Busby et al., 2017; Sheen et al., 2017). Mutation T > C at position 347 and insertion of G between 389 and 390 had inconsistent PZase results in former studies (Aono et al., 2014; Ramirez-Busby and Valafar, 2015). The rest of the mutations showed consistent results related to PZA resistance (Supplementary Table S1). Effects of 30 of the 310 isolates with previously reported mutations on PZase activities had not been determined (Table 4).

**TABLE 4:** Prediction of PZA susceptibilities and PZase activities in 310 isolates by previously reported literatures and their distribution ratios in MDR and XDR TB isolates.

**PZA susceptibility**	**Ratio (%)**	**PZase activity**	**Ratio (%)**
Resistant	294	Negative	273
R/MDR	211/310 (68.1%)	N/MDR	196/310 (63.2%)
R/XDR	67/76 (88.2%)	N/XDR	62/76 (81.6%)
Sensitive	4	Positive	2
ND	2	ND	30
Inconsistent results	10	Inconsistent results	5
Total	310		

**R, resistant to PZA more than 100 μg/ml; N, negative; ND, not determined.**

### Correlation of Novel *pncA* Mutations to PZase Activity

**FIGURE 1:**
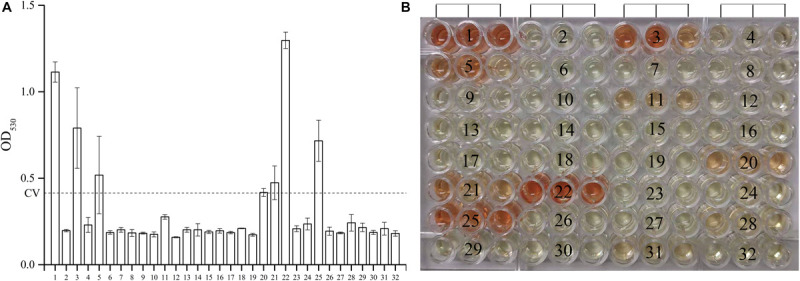
Effects of the 30 new mutations on the enzymatic activity of *Mycobacterium tuberculosis* PncA. **(A)** Measurements of OD_530_ of different wells containing different recombinant *E. coli* W3110Δ*pncA* strains. **(B)** Original color of different wells in 96-well plate. (1) *Escherichia coli* W3110Δ*pncA* pCA24N-*pncA* (positive control); (2) *E.coli* W3110Δ*pncA* pCA24N (negative control); (3) 500C > T (positive); (4) 274G > C (negative); (5) 374T > C (positive); (6) 176C > A and 254T > C (negative); (7) 403_413delACCGATCATTG (negative); (8) 380_381insGG (negative); (9) 50_51insC (negative); (10) 21_27delCGACGTG (negative); (11) 278T > C (negative); (12) 29_37delAGAACGACT (negative); (13) 240_241insT (negative); (14) 201_202insT (negative); (15) 416_417insG (negative); (16) 257A > G and 538G > T (negative); (17) 412T > G (negative); (18) 407_408insTCATTGTGTGCGCCAGACGGC (negative); (19) 470_471insGG (negative); (20) 500_501insCACCGT (positive); (21) 176C > A (positive); (22) 396T > G (positive); (23) 136delG (negative); (24) 520delG (negative); (25) 200C > G (positive); (26) 464_465insG (negative); (27) 137_138insC (negative); (28) 491_492insC (negative); (29) 521_522insT (negative); (30) 425C > G (negative); (31) 232G > C (negative); (32) 310A > G and 388G > A (negative). Every result was shown by test using three independent colonies (mean ± SD); OD, optical density; CV, cutoff value (dash line).

As shown in Figure 1, among the 30 novel mutations, 24 were PZase activity negative and six were positive. Mutations at positions 500C > T, 374T > C, 500_501insCACCGT, 176C > A, 396T > G, and 200C > G maintained their activities. Mutation 396T > G had the highest PZase activity which was even more than that of the positive control. Activity at mutation position 500_501insCACCGT was slightly higher than that of the negative control. All the new mutations without enzyme activity had the values close to that of the negative control. Detailed values of enzyme activity are listed in “Supplementary Dataset S1.”

### Correlation of PZase Activity to PZA Susceptibility

Seventeen isolates (15 PZase negative and 2 positive) with novel *pncA* mutations were subjected to PZA drug susceptibility test by using H37Rv as control. The PZA MIC of H37Rv was determined to be 12.5–25 μg/ml. The isolates with PZase-negative mutations 176C > A and 254T > C, 403_413delACCGATCATTG, 380_381insGG, 50_51insC, 21_27delCGACGTG, 240_241insT, 416_417insG, 257A > G and 538G > T, 407_408insTCATTGTGTGCGCCAGACGGC, 136delG, 520delG, 464_465insG, 521_522insT, 232G > C, and 310A > G and 388G > A, all showed PZA resistance phenotype. On the contrary, the two isolates with PZase-positive mutations 500_501insCACCGT and 396T > G were all shown to be PZA sensitive. Thus, the PZA DST results of all 17 isolates tested were shown to be well consistent with the PZase activity test using our *E. coli* gene knockout strain (Table 5).

**TABLE 5:** Correlation of PZase activity to PZA susceptibility in 17 isolates with novel mutations.

**Strains**	**PncA mutation**		**PZase activity**	**MIC of PZA (μg/ml)**	**No. of isolates**
	**Nucleotide**	**Amino acid**			
H37Rv	No mutation			12.5–25	
3	500C > T	Thr167Ile	Positive	ND	1
4	274G > C	Ala92Pro	Negative	ND	1
5	374T > C	Val125Ala	Positive	ND	1
6	176C > A^*a*^ and 254T > C	Ser59Tyr and Leu85Pro	Negative	>400	1
7	403_413delACCGATCATTG	Frameshift	Negative	>400	2
8	380_381insGG	Frameshift	Negative	>400	1
9	50_51insC	Frameshift	Negative	>400	1
10	21_27delCGACGTG	Frameshift	Negative	>400	1
11	278T > C	Val93Ala	Negative	ND	1
12	29_37delAGAACGACT	Frameshift	Negative	ND	1
13	240_241insT	Frameshift	Negative	>400	1
14	201_202insT	Frameshift	Negative	ND	1
15	416_417insG	Frameshift	Negative	200	3
16	257A > G^*a*^ and 538G > T	Asp86Gly and Val180Phe	Negative	100	1
17	412T > G	Cys138Gly	Negative	ND	1
18	407_408insTCATTGTGTGCGCCAGACGGC	Frameshift	Negative	>400	1
19	470_471insGG	Frameshift	Negative	ND	1
20	500_501insCACCGT	Frameshift	Positive	<12.5	1
21	176C > A	Ser59Tyr	Positive	ND	3
22	396T > G	Gly132Gly	Positive	25	1
23	136delG	Frameshift	Negative	>400	1
24	520delG	Frameshift	Negative	>400	2
25	200C > G	Ser67Trp	Positive	ND	1
26	464_465insG	Frameshift	Negative	>400	1
27	137_138insC	Frameshift	Negative	ND	1
28	491_492insC	Frameshift	Negative	ND	1
29	521_522insT	Frameshift	Negative	>400	1
30	425C > G	Thr142Arg	Negative	ND	1
31	232G > C	Gly78Arg	Negative	>400	1
32	310A > G^*a*^ and 388G > A^*a*^	Ser104Gly and Val130Met	Negative	>400	1

**ND, not done. ^*a*^Novel mutation existed in strains with double mutations in pncA.**

## Discussion

Because of the vital role for the treatment of non-replicative *M. tuberculosis* and shortening the time course of chemotherapy, PZA is contained in traditional treatment for drug-sensitive TB and in modern therapy to treat MDR and XDR TB. Recently, several clinical studies have been carried out for treatment of drug-sensitive and MDR TB with the combination of PZA, PA824, moxifloxacin (Dawson and Diacon, 2013), and bedaquiline (Diacon et al., 2012). Although PZA has been used for decades, it is shocking that the precise mechanism of PZA action is still obscure. One reason is that the action of PZA relies on acidic environment which results in not so well growing of the bacteria. This acidic condition also leads to unreliable results of PZA susceptibility tests. The inaccurate susceptibility results of PZA complicated the research of its mechanism of action and caused improper treatment of TB and spread of PZA resistance. Presently, bacteria culture-based PZA susceptibility tests are usually not performed in most of the hospitals worldwide. Therefore, alternative methods are required. Given that PZA resistance is frequently related to *pncA* mutations (Hirano et al., 1997) and PZase activity (Aono et al., 2014), it makes direct sequencing of *pncA* gene (including its upstream region) and PZase tests good choices. However, not all *pncA* mutations can cause PZA resistance, which weakens the potential of extensive utilization of direct gene sequencing. On the other hand, all PZase methods based on Wayne’s work require large amount of bacterial cultures and thus have to be performed in BSL-3 labs. Therefore, an *in vitro* PZase analysis following *pncA* amplification will be very attractive. Especially, with a continuously updated database containing the full spectrum of *pncA* mutations and the corresponding pyrazinamidase activities, PZA susceptibility tests may not be that difficult any more.

In order to investigate the prevalence of PZA resistance in *M. tuberculosis* isolated from Chongqing district, a total of 465 clinical isolates (including 31 pan-susceptible, 19 mono-resistant, 310 MDR, 76 XDR, and 29 other strains) were collected. Subsequently, the *pncA* gene was amplified, sequenced, and analyzed. The results showed that *pncA* mutations were identified in 346 isolates. We found that *pncA* polymorphisms were presented in 254 (81.9%) MDR and 74 (97.4%) XDR isolates (Table 3). The proportions of *pncA* mutational strains were higher than those from South Africa (70% in MDR, 96% in XDR), Georgia (72% in MDR, 90% in XDR) (Allana et al., 2017), Kazakhstan (75% in MDR) (Akhmetova et al., 2015), and Ukraine (78% in MDR, 88% in XDR) (Daum et al., 2019). We also found that predicted PZA-resistant isolates based on previous literatures in Chongqing district accounts for 68.1% in MDR isolates (Table 4), which was higher than those from Beijing (57.7% in MDR) (Gu et al., 2016), Zhejiang (43.1% in MDR) (Xia et al., 2015), and Taiwan (42.9% in MDR) (Huang et al., 2003). We ascribe this to samples coming from a specific geographic area. Resistance to first-line drugs increased PZA usage. Most of the patients received empirical PZA treatment without the susceptibility tests and increased the possibility of PZA resistance. Otherwise, samples may belong to retreat cases. Former study illustrated that new cases had lower PZA resistance possibility compared with retreat cases (Gu et al., 2016). Additionally, some predictive resistant isolates were borderline resistant at the concentration of 100 μg/ml. The existence of low-level resistance may indicate other mechanism of resistance to PZA, such as an efflux pump involved in the uptake of PZA. Low-level resistance to PZA simultaneously harbored *pncA* mutations. However, whether the resistance was caused by the mutations is uncertain. Considering the high correlation of PZA resistance and MDR, routine assessment of PZA susceptibility would be necessary.

Among the 346 isolates containing *pncA* mutations in this study, 294 isolates were predicted to be PZA resistant according to previous publications (Supplementary Table S4).

Except for those mutations previously reported, 30 new mutations were identified in this study (Table 5). Most of them were deletion or insertion of various lengths of nucleotides. The new mutations scattered between nucleotide position 21–521 (Table 5). Some of the substitutional new mutations, 176C > A, 232G > C, 412T > G, and 425C > G occurred at previously reported nucleotide positions. Mutation 176C > T have been shown to be sensitive to PZA at the concentration of 100 μg/ml (Xia et al., 2015). Our results revealed that the PZase activity of the new mutation 176C > A is positive (Figure 1), suggesting the corresponding clinical isolate is sensitive to PZA. Mutations 232G > T (Somoskovi et al., 2007), 232G > A (Ando et al., 2010), 412T > C (Miyagi et al., 2004), and 425C > T (Scorpio et al., 1997) had been shown to cause PZA resistance. Our results also showed that new mutations 232G > C, 412T > G, and 425C > G also led to loss of PZase activity, suggesting the corresponding clinical isolates are resistant to PZA.

Many other new mutations occurred at previously reported different nucleotides but same amino acid positions. We found that strains with mutations in 50_51insC (Gly17), 136delG (Ala46), 201_202insT (Ser67-Trp68), 240_241insT (Asp80-Phe81), 278T > C (Val93Ala), 310A > G (Ser104Gly), 380_381insGG (Glu127), 388G > A (Val130Met), 464_465insG (Val155), 470_471insGG (Val157), 491_492insC (Ser164), 520delG (Glu174), and 521_522insT (Glu174) were all shown to be PZase negative. Previously, strains with mutations at these amino acid positions, Gly17Asp (Daum et al., 2019), Ala46Val (Cheng et al., 2000), Ser67Pro (Hameed et al., 2020), Trp68Arg (Khan et al., 2019), Asp80Asn (Bwalya et al., 2018), Phe81Val (Wu et al., 2019), Val93Met (Daum et al., 2019), Ser104Ile (Hameed et al., 2020), Glu127Lys (Doustdar et al., 2009), Val130Gly (Sengstake et al., 2017), Val155Ala (Daum et al., 2019), Val157Gly (Khan et al., 2019), Ser164Pro (Hameed et al., 2020), and Glu174Gly (Tan et al., 2014), were reported to be resistant to PZA. PZase-positive mutations, 200C > G (Ser67Trp), 374 T > C (Val125Ala), 396T > G (Gly132Gly), 500C > T (Thr167Ile), and 500_501insCACCGT (Thr167) were identified in five isolates (one isolate for each mutation). Among them, 396T > G (Gly132Gly) is a synonymous mutation. Previous studies also showed the presence of mutations (Ser67Pro and Val125) in PZA susceptible isolates (Allix-Béguec et al., 2018).

Among the 30 isolates with novel *pncA* mutations, 17 of them were successfully recovered and then subjected to PZA DST, and the data showed that the results of PZA DST test were completely consistent to those of the PZase activity tests using the *E. coli* Δ*pncA* strain (Figure 1 and Table 5).

We also tried to predict PZA susceptibilities of isolates with novel single mutations using the SUSPECT-PZA webserver^3^ (Karmakar and Rodrigues, 2020). The prediction results (Supplementary Table S3) were in well consistency with our results, except for mutation 274G > C (sample 4 in Figure 1, Ala92Pro), mutation 278T > C (sample 11, Val93Ala), and mutation 374T > C (sample 5, Val125Ala). From Figure 1, Supplementary Data Sheet S1, and Table 5, we could see that the PZase activity of the mutant 374T > C (Val125Ala) was comparable with that of the 500_501insCACCGT mutant. The PZase activity of 500_501insCACCGT mutant was determined to be positive, and the strain containing the 500_501insCACCGT mutation (K2501) was determined to be sensitive to PZA. However, though the PZase activity of the 278T > C mutant was determined to be negative according to our standard, we did observe residual PZase activity of the mutant (Figure 1 and Supplementary Dataset S1). Unfortunately, the strain with this specific mutation could not be recovered from the refrigerator. We speculated that mutation 278T > C might cause borderline PZA resistance, which needs to be handled with extra caution.

Based on the crystal structure of *M. tuberculosis* PncA, it has been reported that three major regions (amino acids position 3–17, 61–85, and 132–142) are most commonly associated with PZase activity changes (Mehmood et al., 2019), and other regions (amino acids position 15–20, 50–70, 85–100, 130–140, and 160–180) are related to PncA flexibility (Khan and Malik, 2019). New mutations that caused PZase-negative phenotype in this study, mutations 21_27delCGACGTG (Val7-Val9) and 50_51insC (17Gly), are located in amino acid regions 3 to 17. Mutations 201_202insT (Ser67-Trp68) and 240_241insT (Asp80-Phe81) are located in amino acid regions 61 to 85. Mutations 257A > G (Asp86Gly), 274G > C (Ala92Pro), and 278T > C (Val93Ala) are located in amino acid regions 85–100. Mutation 388G > A (Val130Met) is located in amino acid regions 130–140. Mutations 491_492insC (Ser164), 520delG (Glu174), and 521_522insT (Glu174) are located in regions 160–180. So, it is not surprising to see that those mutations lead to the loss of the PZase activity.

Although researches on the mechanisms of PZA action and resistance are still underway, great improvement have been made. Except for *pncA*, more genes have been found to be related with PZA resistance, such as *rpsA*, *panD*, *clpC1*, *rv1411c*, and *rv0521* (Shi et al., 2018). However, mutations in *pncA* still remain the leading cause of PZA resistance. According to previous publications, PZase activity and PZA susceptibility are very closely related. Conventional culture-based methods for PZA susceptibility are often unreliable and time consuming. Therefore, indirect method such as *pncA* gene sequencing and PZase tests would be better choices. To achieve this, a comprehensive database of *pncA* mutations with PZase activities is required. In this study, we not only discovered 30 new *pncA* mutations in *M. tuberculosis* clinical isolates but also developed a new method for PZAse activity test, which will accelerate the utilization of indirect methods for PZA susceptibility tests.

## Data Availability Statement

The original contributions presented in the study are included in the article/Supplementary Material, further inquiries can be directed to the corresponding author/s.

## Ethics Statement

This study used archived strains isolated from clinical samples, no human subjects and no identifiable human data were included. The institutional Ethics Committee of Chongqing Public Health Medical Center approved this study (Permit Number: 2017-CQGW-001-KY) and the exemption from written informed consent had been acquired.

## Author Contributions

JD and YC conceived the idea of study and designed the methodology. ZY and ML prepared all the isolates. KL and JG carried out the experiments. KL wrote the manuscript. All authors contributed to the article and approved the submitted version.

## Conflict of Interest

The authors declare that the research was conducted in the absence of any commercial or financial relationships that could be construed as a potential conflict of interest.

## Footnotes

1 https://blast.ncbi.nlm.nih.gov/Blast.cgi

2 http://varnomen.hgvs.org/

3 http://biosig.unimelb.edu.au/suspect_pza/
