# Supplementary material for: Characterization of pncA Mutations and Prediction of PZA Resistance in Mycobacterium tuberculosis Clinical Isolates From Chongqing, China
Source: Front Microbiol. 2021 Jan 11;11:594171. doi: 10.3389/fmicb.2020.594171 (PMC7832174; doi:10.3389/fmicb.2020.594171)
Supplement: Supplementary file 1 [file Table_2.DOCX]

Table S1 Information of samples used in this study

| Isolates | sampling time(m/d/yr) | Samples ID | Gender | Age(yr) | Sample type | *pncA* mutation | Main associated drug  resistance | Drug susceptibility profile | PZase  activity | PZA susceptibility |
| --- | --- | --- | --- | --- | --- | --- | --- | --- | --- | --- |
|  | 03/27/2017 | F461 | Male | 51 | Sputum | 395G>A | SIRELAC | XDR | N | R |
|  | 06/08/2015 | K3270 | Male | 32 | Sputum | WT | WT | Pan-susceptible |  |  |
|  | 07/24/2015 | K3539 | Female | 37 | Sputum | 407_408insA | SIR | MDR | N | R |
|  | 08/07/2015 | K3604 | Female | 48 | Sputum | WT | SIROL | MDR |  |  |
|  | 08/05/2015 | E903 | Female | 23 | Other | 407_408insA | SIRELC | XDR | N | R |
|  | 02/13/2017 | K7052 | Male | 40 | Sputum | WT | SR | Other |  |  |
|  | 03/02/2015 | K2657 | Female | 47 | Sputum | 395G>A | SIROLA | XDR | N | R |
|  | 09/09/2016 | K6079 | Male | 16 | Sputum | 395G>A | SIRO | MDR | N | R |
|  | 11/20/2015 | k4208 | Female | 36 | Sputum | 395G>A | SIR | MDR | N | R |
|  | 07/01/2014 | E406 | Female | 48 | Fiberoptic bronchoscopy lavage fluid | WT | SIRE | MDR |  |  |
|  | 12/28/2016 | K6842 | Female | 41 | Fiberoptic bronchoscopy lavage fluid | 389_390insGG | SIRM | MDR | N | R |
|  | 06/26/2015 | K3361 | Male | 47 | Sputum | 407_408insA | SIREOLM | MDR | N | R |
|  | 11/27/2015 | K4248 | Male | 35 | Sputum | 523A>G | SIRA | MDR | N | R |
|  | 06/08/2015 | K3273 | Male | 34 | Sputum | WT | WT | Pan-susceptible |  |  |
|  | 03/02/2015 | K2666 | Male | 41 | Sputum | 407_408insA | SIROM | MDR | N | R |
|  | 01/20/2017 | K6956 | Male | 66 | Sputum | WT | SI | Other |  |  |
|  | 09/09/2016 | K6090 | Male | 41 | Sputum | 470_471insGG | SIEOAC | Other |  |  |
|  | 05/26/2016 | F122 | Male | 42 | Sputum | 407_408insA | SIEL | Other | N | R |
|  | 04/25/2014 | E260 | Male | 40 | Sputum | 427G>C | SIREL | MDR | ND | R |
|  | 07/29/2016 | K5819 | Female | 33 | Sputum | 20T>G | SILM | other | N | R |
|  | 12/04/2015 | K4324 | Male | 41 | Sputum | WT | SIRLA | XDR |  |  |
|  | 06/01/2014 | E358 | Male | 59 | Other | 41G>A | SIREACL | XDR | N | R |
|  | 06/08/2015 | K3276 | Male | 26 | Sputum | WT | WT | Pan-susceptible |  |  |
|  | 09/25/2015 | E941 | Female | 26 | Sputum | 407_408insA | SIREL | MDR | N | R |
|  | 10/15/2015 | E945 | Male | 52 | Other | 523A>G | SIREL | MDR | N | R |
|  | 05/07/2014 | E285 | Male | 49 | Sputum | 28C>T | SIRECL | XDR | N | R |
|  | 10/24/2016 | K6407 | Male | 46 | Sputum | 389_390insGG | SIR | MDR | N | R |
|  | 06/03/2015 | K3243 | Male | 47 | Sputum | 395G>A | SIR | MDR | N | R |
|  | 01/29/2015 | K2543 | Female | 28 | Sputum | 407_408insA | SIRA | MDR | N | R |
|  | 09/25/2015 | E940 | Male | 28 | Fiberoptic bronchoscopy lavage fluid | 395G>A | SREL | Other | N | R |
|  | 11/06/2015 | K4110 | Male | 39 | Sputum | 40T>C | SIROLAM | XDR | N | R |
|  | 02/05/2016 | K4643 | Male | 43 | Sputum | 241T>G | IROA | XDR | ND | R |
|  | 06/08/2015 | K3253 | Male | 47 | Pyogenic fluid | 407_408insA | SIRA | MDR | N | R |
|  | 01/11/2017 | K6890 | Female | 31 | Sputum | 20T>G | S | Mono | N | R |
|  | 02/13/2017 | K7059 | Male | 45 | Sputum | 409C>G | IRA | MDR | N | R |
|  | 10/15/2014 | E578 | Male | 43 | Sputum | 407_408insA | SIREL | MDR | N | R |
|  | 04/27/2015 | E828 | Male | 37 | Sputum | 355T>C | SIRE | MDR | ND | R |
|  | 06/08/2015 | K3280 | Male | 16 | Fiberoptic bronchoscopy lavage fluid | 463_464insT | SIR | MDR | N | ND |
|  | 12/29/2016 | F328 | Male | 45 | Sputum | 406G>T | SIREACL | XDR | N | R |
|  | 12/04/2015 | K4287 | Male | 63 | Sputum | 20T>G | SIR | MDR | N | R |
|  | 06/17/2016 | K5517 | Female | 52 | Sputum | 278T>C | SIREOLM | MDR |  |  |
|  | 10/30/2015 | E959 | Male | 45 | Other | 185C>T | SIREACL | XDR | N | R |
|  | 01/21/2015 | K2501 | Male | 52 | Sputum | 500_501insCACCGT | IROAM | XDR |  |  |
|  | 10/14/2015 | K4012 | Male | 32 | Sputum | 395G>A | IR | MDR | N | R |
|  | 12/04/2015 | K4275 | Female | 26 | Sputum | -12T>C | SIRA | MDR | N | R |
|  | 10/30/2015 | E958 | Male | 23 | Other | 40T>C | SIREACL | XDR | N | R |
|  | 12/18/2015 | K4373 | Female | 41 | Sputum | WT | SIR | MDR |  |  |
|  | 09/19/2016 | K6115 | Male | 41 | Sputum | 394G>A | SIRE | MDR | N | R |
|  | 07/21/2015 | K3513 | Male | 50 | Sputum | 395G>T | SIR | MDR | N | R |
|  | 05/05/2015 | K3058 | Male | 18 | Sputum | WT | IE | Other |  |  |
|  | 11/06/2015 | K4099 | Female | 30 | Sputum | 407_408insA | SIROAM | XDR | N | R |
|  | 12/29/2016 | F330 | Female | 27 | Fiberoptic bronchoscopy lavage fluid | 395G>A | SIRE | MDR | N | R |
|  | 06/08/2015 | K3283 | Female | 72 | Sputum | 143A>C | SIRO | MDR | ND | ? |
|  | 07/27/2015 | E901 | Male | 29 | Sputum | 35A>G | SIREL | MDR | N | ? |
|  | 12/04/2015 | K4279 | Male | 29 | Pyogenic fluid | 407_408insA | SIR | MDR | N | R |
|  | 07/24/2017 | K8280 | Female | 42 | Sputum | 40T>C | SIRLACM | XDR | N | R |
|  | 08/14/2017 | K8534 | Male | 19 | Sputum | 407_408insA | SIR | MDR | N | R |
|  | 07/24/2017 | K8315 | Male | 29 | Sputum | 407_408insA | SIRLA | XDR | N | R |
|  | 02/02/2016 | K4603 | Male | 50 | Sputum | 322G>C | SIROLM | MDR | N | R |
|  | 10/25/2017 | K9112 | Female | 29 | Fiberoptic bronchoscopy lavage fluid | 395delG | SIR | MDR | ND | R |
|  | 08/24/2017 | K8616 | Male | 52 | Sputum | 403_413delACCGATCATTG | IR | MDR |  |  |
|  | 05/26/2016 | F123 | Male | 27 | Sputum | 464_465insG | SIREL | MDR |  |  |
|  | 09/28/2017 | K8941 | Male | 27 | Sputum | 171C>G | SIR | MDR | N | R |
|  | 07/24/2017 | K8301 | Female | 48 | Sputum | 407_408insA | SIRA | MDR | N | R |
|  | 10/18/2016 | F241 | Male | 42 | Sputum | 515T>G | SIREACL | XDR | ND | R |
|  | 08/14/2017 | K8426 | Male | 48 | Sputum | WT | IR | MDR |  |  |
|  | 04/28/2017 | K7678 | Male | 18 | Sputum | 407_408insA | SIR | MDR | N | R |
|  | 12/04/2015 | K4303 | Female | 38 | Sputum | 139A>G | SIRA | MDR | N | ? |
|  | 11/02/2017 | F859 | Female | 31 | Fiberoptic bronchoscopy lavage fluid | 389T>G | SIREACL | XDR | N | R |
|  | 03/16/2016 | K4848 | Male | 39 | Sputum | WT | IROL | MDR |  |  |
|  | 02/26/2016 | K4701 | Male | 56 | Sputum | WT | RL | Other |  |  |
|  | 03/16/2016 | K4852 | Male | 38 | Sputum | 226A>C | SIR | MDR | N | R |
|  | 02/14/2017 | F391 | Male | 31 | Sputum | 17T>C | SIRE | MDR | N | R |
|  | 08/14/2017 | K8500 | Male | 31 | Sputum | WT | I | Mono |  |  |
|  | 10/25/2017 | K9133 | Male | 65 | Sputum | 395G>A | SIR | MDR | N | R |
|  | 03/30/2015 | E789 | Male | 57 | Sputum | 123C>G | SIREACL | XDR | ND | R |
|  | 10/17/2017 | K9037 | Male | 28 | Sputum | 226A>C | SIRM | MDR | N | R |
|  | 07/24/2017 | K8311 | Male | 57 | Sputum | 274G>C | SIR | MDR |  |  |
|  | 09/30/2014 | E576 | Female | 37 | Sputum | 395G>A | SIRE | MDR | N | R |
|  | 08/31/2017 | K8714 | Male | 45 | Sputum | 56T>G | SIR | MDR | N | R |
|  | 02/26/2016 | K4730 | Male | 69 | Sputum | 389T>G | SIM | Other | N | R |
|  | 04/28/2017 | K7674 | Male | 48 | Sputum | 226A>C | SIR | MDR | N | R |
|  | 10/04/2017 | F810 | Male | 43 | Sputum | 407_408insA | SIREACL | XDR | N | R |
|  | 03/27/2017 | F462 | Male | 52 | Sputum | 395G>A | SIREACL | XDR | N | R |
|  | 09/24/2017 | K8902 | Female | 21 | Pleural fluid | 467T>C | SIRA | MDR | ND | R |
|  | 01/16/2015 | K2481 | Female | 38 | Fiberoptic bronchoscopy lavage fluid | 395G>A | SIRO | MDR | N | R |
|  | 03/03/2017 | K7234 | Female | 24 | Sputum | 545T>G | SIR | MDR | ND | R |
|  | 10/18/2017 | F827 | Male | 45 | Sputum | -11A>G | SIREL | MDR | N | R |
|  | 08/30/2017 | F737 | Female | 62 | Other | 407_408insA | SIREACL | XDR | N | R |
|  | 09/13/2017 | K8835 | Female | 21 | Sputum | 322G>C | SIRA | MDR | N | R |
|  | 02/26/2016 | K4702 | Male | 41 | Sputum | 407_408insA | SIROLM | MDR | N | R |
|  | 10/13/2017 | F812 | Female | 30 | Sputum | 407_408insA | SIRECL | XDR | N | R |
|  | 03/16/2016 | K4854 | Male | 21 | Cerebrospinal fluid | 520delG | SIR | MDR |  |  |
|  | 10/08/2017 | K8995 | Male | 60 | Sputum | WT | SI | Other |  |  |
|  | 12/26/2014 | K2403 | Female | 25 | Sputum | 407_408insA | SIRO | MDR | N | R |
|  | 08/14/2017 | K8497 | Male | 29 | Sputum | 515T>C | SIR | MDR | N | R |
|  | 05/22/2015 | K3181 | Male | 49 | Fiberoptic bronchoscopy lavage fluid | WT | WT | Pan-susceptible |  |  |
|  | 08/14/2017 | K8435 | Male | 46 | Sputum | 407_408insA | SIR | MDR | N | R |
|  | 07/24/2017 | K8286 | Female | 13 | Sputum | 17T>C | SIRAC | MDR | N | R |
|  | 02/05/2016 | K4620 | Male | 63 | Sputum | WT | SIRL | MDR |  |  |
|  | 01/27/2016 | K4571 | Male | 33 | Sputum | 395G>A | SIR | MDR | N | R |
|  | 08/14/2017 | K8433 | Male | 63 | Sputum | 185C>T | SIR | MDR | N | R |
|  | 08/14/2017 | K8522 | Male | 16 | Sputum | 407A>G | IRAC | MDR | N | R |
|  | 07/24/2017 | K8313 | Male | 51 | Sputum | WT | IR | MDR |  |  |
|  | 04/28/2017 | K7672 | Female | 30 | Fiberoptic bronchoscopy lavage fluid | 407_408insA | SIR | MDR | N | R |
|  | 08/24/2017 | K8646 | Male | 21 | Sputum | 395G>A | SIR | MDR | N | R |
|  | 08/26/2016 | K5970 | Male | 46 | Sputum | WT | SIRO | MDR |  |  |
|  | 04/28/2017 | K7664 | Male | 20 | Sputum | 226A>C | SIR | MDR | N | R |
|  | 09/19/2016 | K6147 | Male | 66 | Sputum | 407_408insA | SIROA | XDR | N | R |
|  | 01/29/2015 | K2553 | Female | 21 | Pyogenic fluid | WT | SIR | MDR |  |  |
|  | 03/05/2015 | K2698 | Male | 47 | Sputum | 407_408insA | SIR | MDR | N | R |
|  | 02/16/2016 | K4676 | Female | 33 | Sputum | 407_408insA | SIREOLM | MDR | N | R |
|  | 08/31/2017 | K8708 | Male | 61 | Sputum | 380_381insGG | SIRLM | MDR |  |  |
|  | 03/09/2016 | K4803 | Male | 39 | Sputum | 226A>C | SIR | MDR | N | R |
|  | 10/08/2017 | K9004 | Male | 67 | Sputum | 71G>A | SIR | MDR | ND | R |
|  | 08/02/2017 | K8360 | Male | 51 | Sputum | 416_417insG | SIRLACM | XDR |  |  |
|  | 07/24/2017 | K8350 | Male | 40 | Sputum | WT | SIR | MDR |  |  |
|  | 06/08/2015 | K3266 | Female | 45 | Sputum | 347T>C | SIROM | MDR | ? | R |
|  | 07/09/2015 | K3443 | Female | 34 | Fiberoptic bronchoscopy lavage fluid | WT | SIR | MDR |  |  |
|  | 07/21/2015 | K3504 | Male | 52 | Sputum | 35A>C | SIROLM | MDR | N | R |
|  | 04/12/2017 | F483 | Male | 20 | Sputum | 436G>A | SIREACL | XDR | N | R |
|  | 03/04/2016 | K4766 | Female | 27 | Sputum | 35A>G | SIR | MDR | N | ? |
|  | 08/14/2015 | K3665 | Female | 67 | Sputum | WT | IR | MDR |  |  |
|  | 02/17/2017 | K7222 | Male | 52 | Sputum | 193T>C; 359T>C | SIR | MDR | ? | R |
|  | 10/14/2016 | K6328 | Female | 31 | Sputum | 389_390insGG | SIRM | MDR | N | R |
|  | 03/25/2016 | K4944 | Female | 43 | Sputum | 257A>G; 538G>T | SIR | MDR |  |  |
|  | 06/12/2015 | K3290 | Female | 27 | Sputum | WT | O | Mono |  |  |
|  | 07/10/2015 | E891 | Female | 31 | Sputum | 422A>C | SIREACL | XDR | N | R |
|  | 11/28/2016 | K6599 | Male | 30 | Sputum | WT | SIR | MDR |  |  |
|  | 11/14/2016 | K6523 | Female | 26 | Pyogenic fluid | WT | SIR | MDR |  |  |
|  | 10/31/2016 | K6457 | Male | 20 | Sputum | WT | SIR | MDR |  |  |
|  | 01/06/2017 | K6850 | Male | 56 | Sputum | WT | WT | Pan-susceptible |  |  |
|  | 11/13/2015 | K4160 | Male | 31 | Sputum | 11T>G | SIR | MDR | N | ND |
|  | 05/11/2016 | F113 | Female | 42 | Fiberoptic bronchoscopy lavage fluid | 407_408insA | SIREL | MDR | N | R |
|  | 10/24/2016 | K6415 | Female | 22 | Sputum | 188A>G | SIRAM | XDR | N | R |
|  | 05/15/2015 | E843 | Male | 59 | Sputum | WT | SIREL | MDR |  |  |
|  | 01/26/2017 | K7009 | Female | 63 | Pyogenic fluid | WT | WT | Pan-susceptible |  |  |
|  | 06/10/2015 | E862 | Male | 41 | Sputum | 416_417insG | SIREL | MDR |  |  |
|  | 10/15/2015 | E944 | Male | 45 | Other | WT | SIREL | MDR |  |  |
|  | 07/24/2015 | K3538 | Female | 17 | Sputum | 143A>C | SIRM | MDR | ND | ? |
|  | 11/30/2014 | E659 | Female | 25 | Fiberoptic bronchoscopy lavage fluid | 407_408insA | SIREACL | XDR | N | R |
|  | 01/20/2017 | K6954 | Male | 16 | Sputum | 226A>C | SR | Other | N | R |
|  | 01/20/2017 | K6927 | Male | 46 | Sputum | WT | WT | Pan-susceptible |  |  |
|  | 02/09/2015 | K2578 | Male | 39 | Sputum | 416_417insG | SIROLM | MDR |  |  |
|  | 06/12/2015 | K3295 | Female | 18 | Sputum | 20T>G | SIRE | MDR | N | R |
|  | 09/15/2014 | E515 | Female | 66 | Sputum | WT | I | Mono |  |  |
|  | 09/19/2016 | K6138 | Male | 24 | Pleural fluid | WT | IE | Other |  |  |
|  | 08/22/2016 | K5967 | Male | 57 | Pleural fluid | WT | I | Mono |  |  |
|  | 09/21/2016 | K6195 | Male | 37 | Sputum | 35A>G | SIRO | MDR | N | ? |
|  | 06/03/2015 | K3229 | Female | 35 | Sputum | 395G>A | SIR | MDR | N | R |
|  | 10/31/2016 | K6454 | Male | 45 | Cerebrospinal fluid | 176C>T | SI | Other | ND | S |
|  | 11/07/2016 | K6487 | Male | 28 | Sputum | WT | SIR | MDR |  |  |
|  | 04/05/2017 | F475 | Male | 69 | Sputum | 395G>A | SIREACL | XDR | N | R |
|  | 12/12/2016 | K6709 | Female | 49 | Sputum | 395G>A | SIRM | MDR | N | R |
|  | 07/15/2014 | E440 | Male | 60 | Sputum | 322G>C | SIREAL | XDR | N | R |
|  | 02/17/2015 | K2614 | Male | 17 | Sputum | 50_51insC | IRAC | MDR |  |  |
|  | 12/25/2014 | K2389 | Male | 45 | Sputum | 287A>G | IOA | Other | N | R |
|  | 08/04/2016 | K5863 | Male | 41 | Sputum | 396T>G | SIROM | MDR |  |  |
|  | 07/15/2014 | E435 | Female | 38 | Sputum | 188A>C | SIR | MDR | N | R |
|  | 01/04/2015 | K2422 | Female | 75 | Sputum | 35A>C | SIROAM | XDR | N | R |
|  | 11/07/2016 | K6480 | Male | 44 | Sputum | 21_27delCGACGTG | SIR | MDR |  |  |
|  | 02/24/2017 | K7226 | Male | 40 | Fiberoptic bronchoscopy lavage fluid | WT | I | Mono |  |  |
|  | 06/03/2015 | K3214 | Female | 59 | Sputum | WT | S | Mono |  |  |
|  | 02/10/2017 | K7210 | Female | 22 | Sputum | WT | IE | Other |  |  |
|  | 02/13/2017 | K7050 | Female | 26 | Fiberoptic bronchoscopy lavage fluid | WT | SIR | MDR |  |  |
|  | 06/08/2015 | K3267 | Male | 47 | Sputum | 407_408insA | SIRA | MDR | N | R |
|  | 10/30/2015 | E960 | Male | 43 | Other | 407_408insA | SIREL | MDR | N | R |
|  | 11/30/2015 | E976 | Male | 40 | Pyogenic fluid | 395G>A | SIREC | MDR | N | R |
|  | 11/20/2015 | K4197 | Male | 30 | Sputum | 407_408insA | SIR | MDR | N | R |
|  | 11/27/2015 | K4239 | Male | 40 | Sputum | 232G>T | SIROLAM | XDR | ND | R |
|  | 01/16/2017 | F336 | Female | 24 | Pyogenic fluid | 395G>A | SIRE | MDR | N | R |
|  | 09/05/2016 | K6030 | Male | 27 | Fiberoptic bronchoscopy lavage fluid | 20T>G | SIRO | MDR | N | R |
|  | 07/27/2016 | K5791 | Male | 26 | Fiberoptic bronchoscopy lavage fluid | 240_241insT | SIRO | MDR |  |  |
|  | 07/30/2014 | E465 | Male | 40 | Sputum | 203G>T | SIRE | MDR | N | R |
|  | 11/09/2017 | K9277 | Female | 63 | Sputum | 395G>A | SIR | MDR | N | R |
|  | 09/08/2016 | F214 | Male | 62 | Sputum | WT | SIREL | MDR |  |  |
|  | 08/25/2017 | F723 | Male | 25 | Other | 308A>G | SIREL | MDR | N | R |
|  | 06/08/2015 | K3262 | Male | 41 | Sputum | WT | SIR | MDR |  |  |
|  | 09/15/2017 | F756 | Female | 68 | Fiberoptic bronchoscopy lavage fluid | 329A>G | SIRE | MDR | ND | R |
|  | 05/29/2015 | E847 | Male | 20 | Fiberoptic bronchoscopy lavage fluid | 35A>C | IREL | MDR | N | R |
|  | 07/01/2014 | E391 | Female | 46 | Sputum | 467T>C | SIREAL | XDR | ND | R |
|  | 01/22/2016 | K4560 | Male | 51 | Sputum | 415G>A | IROL | MDR | N | R |
|  | 01/22/2016 | K4558 | Male | 57 | Sputum | 202T>C | SIROLM | MDR | N | R |
|  | 09/09/2017 | K8790 | Male | 34 | Sputum | WT | IR | MDR |  |  |
|  | 06/01/2017 | F541 | Male | 50 | Sputum | 407_408insA | SIREL | MDR | N | R |
|  | 10/27/2017 | K9160 | Male | 45 | Sputum | 389_390insG | SIR | MDR | N | R |
|  | 02/05/2016 | K4616 | Male | 14 | Fiberoptic bronchoscopy lavage fluid | 20T>G | SIREOLM | MDR | N | R |
|  | 11/30/2016 | F298 | Male | 25 | Sputum | 407_408insA | SIREL | MDR | N | R |
|  | 12/07/2017 | F928 | Male | 22 | Sputum | 185C>T | SIREACL | XDR | N | R |
|  | 05/25/2018 | KA734 | Male | 39 | Sputum | -11A>G | SIR | MDR | N | R |
|  | 05/25/2018 | KA720 | Male | 53 | Sputum | 407_408insA | SIRE | MDR | N | R |
|  | 12/02/2016 | K6640 | Female | 25 | Sputum | 29A>C | SI | Other | N | R |
|  | 12/07/2017 | F926 | Female | 27 | Fiberoptic bronchoscopy lavage fluid | 395G>A | SIRE | MDR | N | R |
|  | 06/14/2018 | 400 | Female | 24 | Sputum | 20T>G | SIREL | MDR | N | R |
|  | 06/01/2018 | KA792 | Male | 21 | Sputum | 412T>C | SIR | MDR | N | R |
|  | 08/19/2016 | F211 | Male | 35 | Sputum | 407_408insA | SIREACL | XDR | N | R |
|  | 04/24/2018 | KA493 | Male | 20 | Sputum | 395G>A | SIR | MDR | N | R |
|  | 01/11/2017 | K6902 | Male | 34 | Sputum | WT | R | Mono |  |  |
|  | 12/20/2017 | K9514 | Female | 42 | Sputum | 187G>C | IRC | MDR | N | R |
|  | 05/17/2018 | KA691 | Female | 50 | Sputum | 20T>C | SIR | MDR | N | R |
|  | 12/12/2016 | K6722 | Male | 38 | Sputum | 146A>C | SIRA | MDR | N | R |
|  | 06/02/2017 | K7807 | Female | 42 | Sputum | 176C>A; 254T>C | IR | MDR |  |  |
|  | 05/10/2018 | KA636 | Female | 22 | Sputum | 407_408insA | SIR | MDR | N | R |
|  | 05/08/2018 | KA629 | Female | 42 | Fiberoptic bronchoscopy lavage fluid | 389_390insGG | SIR | MDR | N | R |
|  | 06/01/2018 | KA779 | Male | 55 | Sputum | 146A>C | SIRA | MDR | N | R |
|  | 07/21/2015 | K3514 | Female | 26 | Sputum | 152A>C | SIROLAM | XDR | N | R |
|  | 07/02/2018 | G432 | Male | 48 | Sputum | 20T>G | SIREL | MDR | N | R |
|  | 03/23/2016 | K4930 | Female | 27 | Sputum | 407_408insA | SIROM | MDR | N | R |
|  | 09/09/2016 | K6105 | Male | 44 | Sputum | -11A>G | SIROAC | XDR | N | R |
|  | 11/22/2017 | F899 | Male | 23 | Sputum | 226A>C | SIREAL | XDR | N | R |
|  | 01/16/2018 | K9674 | Female | 47 | Sputum | 395G>A | SIR | MDR | N | R |
|  | 11/30/2016 | F288 | Male | 18 | Pyogenic fluid | 407_408insA | SIRECL | XDR | N | R |
|  | 04/14/2017 | K7601 | Male | 44 | Sputum | WT | SIR | MDR |  |  |
|  | 03/30/2016 | K4972 | Male | 45 | Sputum | 185C>T | SIR | MDR | N | R |
|  | 01/20/2017 | K6941 | Female | 58 | Sputum | 407_408insA | SIR | MDR | N | R |
|  | 07/08/2016 | K5674 | Female | 35 | Sputum | WT | SIRO | MDR |  |  |
|  | 11/28/2016 | K6568 | Female | 41 | Sputum | 20T>G | S | Mono | N | R |
|  | 05/25/2018 | KA712 | Female | 44 | Sputum | 146A>C | IR | MDR | N | R |
|  | 04/27/2016 | F077 | Male | 24 | Sputum | 40T>C | SIRECL | XDR | N | R |
|  | 05/04/2016 | F98 | Male | 35 | Other | 190T>G | SIRE | MDR | N | R |
|  | 03/16/2016 | K4858 | Male | 54 | Sputum | 185C>T | SIROLM | MDR | N | R |
|  | 07/03/2015 | K3412 | Male | 57 | Pyogenic fluid | WT | WT | Mono |  |  |
|  | 09/08/2016 | F216 | Male | 35 | Sputum | 407_408insA | SIREL | MDR | N | R |
|  | 01/13/2018 | K9669 | Female | 58 | Sputum | 395G>A | SIR | MDR | N | R |
|  | 01/26/2017 | K6987 | Male | 41 | Sputum | 395G>A | SRA | Other | N | R |
|  | 03/01/2018 | KA026 | Male | 38 | Sputum | 291_292insG | IRA | MDR | ND | R |
|  | 03/09/2018 | KA070 | Female | 23 | Sputum | 395G>A | SIR | MDR | N | R |
|  | 01/18/2018 | F970 | Male | 36 | Other | 422A>C | SIREL | MDR | N | R |
|  | 03/23/2018 | KA187 | Female | 27 | Sputum | 389_390insGG | SIR | MDR | N | R |
|  | 05/29/2015 | E853 | Male | 52 | Fiberoptic bronchoscopy lavage fluid | 185C>T | SIREACL | XDR | N | R |
|  | 12/07/2017 | F930 | Male | 44 | Sputum | 395G>A | SIRECL | XDR | N | R |
|  | 01/12/2018 | K9630 | Male | 30 | Sputum | 395G>A | SIR | MDR | N | R |
|  | 04/26/2017 | F506 | Male | 26 | Pleural fluid | 185C>T | SIREACL | XDR | N | R |
|  | 03/02/2018 | G094 | Male | 21 | Sputum | 395G>A | IL | Other | N | R |
|  | 04/05/2018 | G216 | Female | 50 | Sputum | 539T>C | SIREAL | XDR | ND | R |
|  | 08/14/2017 | K8494 | Male | 60 | Sputum | -11A>G | SIR | MDR | N | R |
|  | 07/30/2017 | E456 | Male | 67 | Sputum | 185C>T | SIREAC | MDR | N | R |
|  | 10/27/2017 | K9166 | Male | 42 | Sputum | WT | SIR | MDR |  |  |
|  | 07/15/2016 | F154 | Male | 18 | Sputum | 226A>C | SIREL | MDR | N | R |
|  | 05/08/2018 | KA619 | Female | 30 | Cerebrospinal fluid | 395G>A | SIR | MDR | N | R |
|  | 02/14/2018 | K9930 | Female | 25 | Fiberoptic bronchoscopy lavage fluid | WT | SR | Other |  |  |
|  | 03/15/2017 | F432 | Male | 65 | Sputum | 226A>C | SIREL | MDR | N | R |
|  | 07/19/2018 | G502 | Male | 51 | Sputum | 407A>G | REAL | Other | N | R |
|  | 07/09/2015 | K3459 | Male | 58 | Sputum | 407_408insA | SIRO | MDR | N | R |
|  | 12/23/2016 | K6776 | Male | 33 | Sputum | 389_390insGG | SIR | MDR | N | R |
|  | 03/23/2017 | F440 | Male | 35 | Cerebrospinal fluid | 407_408insA | SIREL | MDR | N | R |
|  | 06/02/2017 | K7805 | Male | 26 | Sputum | 407_408insA | SIR | MDR | N | R |
|  | 03/09/2018 | KA066 | Male | 25 | Pyogenic fluid | 190T>G | SIR | MDR | N | R |
|  | 12/11/2015 | K4354 | Male | 60 | Sputum | 407_408insA | SIRA | MDR | N | R |
|  | 05/15/2015 | E834 | Male | 51 | Fiberoptic bronchoscopy lavage fluid | 187G>C | IREL | MDR | N | R |
|  | 03/13/2018 | KA087 | Female | 15 | Sputum | 395G>A | SIRLAC | XDR | N | R |
|  | 04/08/2018 | KA310 | Female | 49 | Sputum | 309C>G | SIRA | MDR | N | R |
|  | 04/13/2018 | KA387 | Male | 70 | Sputum | 407_408insA | SIREA | MDR | N | R |
|  | 03/06/2018 | KA045 | Male | 43 | Pyogenic fluid | 407_408insA | SIR | MDR | N | R |
|  | 03/20/2018 | KA156 | Male | 64 | Sputum | 407_408insA | SIR | MDR | N | R |
|  | 05/27/2016 | K5366 | Female | 47 | Sputum | 407_408insA | SIREOLAM | XDR | N | R |
|  | 11/30/2014 | E657 | Female | 21 | Sputum | WT | SIREACL | XDR |  |  |
|  | 07/24/2015 | K3526 | Female | 25 | Sputum | 40T>C | SIROLM | MDR | N | R |
|  | 06/01/2017 | F543 | Female | 65 | Sputum | -11A>G | SIREACL | XDR | N | R |
|  | 11/14/2016 | K6533 | Male | 79 | Sputum | WT | WT | Pan-susceptible |  |  |
|  | 05/15/2017 | K7747 | Female | 36 | Sputum | 407_408insA | SIR | MDR | N | R |
|  | 07/03/2015 | K3386 | Male | 18 | Fiberoptic bronchoscopy lavage fluid | WT | WT | Pan-susceptible |  |  |
|  | 07/21/2015 | K3505 | Male | 17 | Pyogenic fluid | WT | WT | Pan-susceptible |  |  |
|  | 08/08/2016 | F172 | Male | 22 | Other | WT | SIREL | MDR |  |  |
|  | 06/26/2015 | K3380 | Male | 23 | Pleural fluid | WT | WT | Pan-susceptible |  |  |
|  | 04/14/2017 | K7500 | Male | 37 | Sputum | WT | I | Mono |  |  |
|  | 03/27/2017 | K7330 | Female | 44 | Sputum | 407_408insA | SIR | MDR | N | R |
|  | 05/15/2017 | K7748 | Female | 52 | Sputum | WT | WT | Pan-susceptible |  |  |
|  | 01/10/2017 | F356 | Female | 21 | Sputum | 232G>A | SIREACL | XDR | N | R |
|  | 06/19/2017 | K8120 | Female | 43 | Other | 174C>A | SIRELACM | XDR | N | R |
|  | 06/16/2017 | K7853 | Male | 64 | Sputum | 146A>C | SIRAC | MDR | N | R |
|  | 06/16/2017 | K7859 | Female | 54 | Sputum | 226A>C | SIR | MDR | N | R |
|  | 06/26/2015 | K3384 | Male | 27 | Sputum | WT | WT | Pan-susceptible |  |  |
|  | 05/15/2017 | K7739 | Female | 23 | Sputum | 20T>G | SI | Other | N | R |
|  | 06/26/2015 | K3369 | Male | 69 | Fiberoptic bronchoscopy lavage fluid | WT | WT | Pan-susceptible |  |  |
|  | 05/27/2016 | K5394 | Male | 65 | Sputum | WT | IRO | MDR |  |  |
|  | 03/27/2017 | K7334 | Male | 21 | Sputum | WT | WT | Pan-susceptible |  |  |
|  | 05/20/2016 | K5356 | Male | 32 | Sputum | 204G>A | IROLM | MDR | N | R |
|  | 05/15/2017 | K7762 | Male | 61 | Sputum | WT | I | Mono |  |  |
|  | 04/26/2018 | G299 | Female | 31 | Pyogenic fluid | 185C>T | SIREL | MDR | N | R |
|  | 05/08/2015 | K3079 | Male | 65 | Sputum | WT | WT | Pan-susceptible |  |  |
|  | 01/08/2018 | F961 | Male | 46 | Sputum | 17T>C | SIREAL | XDR | N | R |
|  | 05/20/2016 | K5354 | Female | 18 | Pyogenic fluid | 521_522insT | IRC | MDR |  |  |
|  | 12/11/2015 | K4358 | Male | 52 | Sputum | WT | OLM | Other |  |  |
|  | 07/24/2015 | K3536 | Male | 44 | Sputum | WT | WT | Pan-susceptible |  |  |
|  | 04/08/2016 | K5032 | Female | 33 | Sputum | WT | IR | MDR |  |  |
|  | 04/28/2017 | K7682 | Male | 63 | Sputum | 359T>G | SIRE | MDR | N | R |
|  | 06/12/2017 | K7985 | Female | 79 | Sputum | WT | SIR | MDR |  |  |
|  | 07/15/2015 | K3475 | Female | 24 | Sputum | WT | WT | Pan-susceptible |  |  |
|  | 07/03/2015 | K3429 | Male | 24 | Fiberoptic bronchoscopy lavage fluid | WT | S | Mono |  |  |
|  | 07/03/2015 | K3401 | Female | 73 | Sputum | WT | WT | Pan-susceptible |  |  |
|  | 03/25/2016 | K4932 | Male | 49 | Sputum | 290G>A | SIROL | MDR | N | R |
|  | 03/27/2017 | K7337 | Female | 60 | Sputum | 347T>C | SRLA | Other | ? | R |
|  | 02/21/2017 | F402 | Female | 27 | Pyogenic fluid | 226A>C | SIREL | MDR | N | R |
|  | 05/20/2016 | K5333 | Male | 63 | Sputum | WT | SIC | Other |  |  |
|  | 06/02/2017 | K7843 | Male | 26 | Sputum | 401C>T | SIR | MDR | N | R |
|  | 05/22/2017 | K7792 | Male | 72 | Sputum | 310A>G;388G>A | SIRLM | MDR |  |  |
|  | 04/07/2017 | K7474 | Male | 51 | Sputum | WT | SIRLM | MDR |  |  |
|  | 04/14/2017 | K7490 | Female | 39 | Sputum | WT | WT | Pan-susceptible |  |  |
|  | 03/18/2016 | K4898 | Male | 62 | Sputum | WT | SIRA | MDR |  |  |
|  | 04/08/2016 | K5016 | Male | 53 | Sputum | WT | I | Mono |  |  |
|  | 05/20/2016 | K5297 | Male | 29 | Sputum | WT | SI | Other |  |  |
|  | 06/22/2016 | F153 | Female | 21 | Sputum | 407_408insTCATTGTGTGCGCCAGACGGC | SIRECL | XDR |  |  |
|  | 05/18/2015 | K3116 | Female | 20 | Pyogenic fluid | WT | WT | Pan-susceptible |  |  |
|  | 06/16/2015 | K3324 | Male | 30 | Pleural fluid | WT | OLM | Other |  |  |
|  | 05/08/2017 | K7586 | Male | 62 | Sputum | 422A>C | SIRA | MDR | N | R |
|  | 02/16/2016 | K4680 | Male | 25 | Sputum | 226A>C | SIREOLAM | XDR | N | R |
|  | 11/22/2017 | F904 | Female | 21 | Other | WT | SIRL | MDR |  |  |
|  | 05/27/2015 | K3204 | Male | 40 | Sputum | 17T>C | SIRA | MDR | N | R |
|  | 06/12/2017 | K7983 | Female | 20 | Sputum | WT | WT | Pan-susceptible |  |  |
|  | 11/22/2017 | F905 | Male | 25 | Pleural fluid | WT | WT | Pan-susceptible |  |  |
|  | 03/29/2018 | G170 | Male | 62 | Sputum | WT | I | Mono |  |  |
|  | 02/06/2017 | K7035 | Male | 69 | Sputum | WT | WT | Pan-susceptible |  |  |
|  | 01/12/2018 | K9585 | Male | 60 | Sputum | WT | WT | Pan-susceptible |  |  |
|  | 09/23/2016 | K6179 | Female | 48 | Sputum | -11A>G | SIROM | MDR | N | R |
|  | 03/23/2018 | KA184 | Female | 63 | Sputum | 359T>G | SI | Other | N | R |
|  | 09/30/2016 | K6215 | Male | 21 | Fiberoptic bronchoscopy lavage fluid | WT | SIO | Other |  |  |
|  | 11/22/2017 | F907 | Male | 55 | Sputum | WT | WT | Pan-susceptible |  |  |
|  | 12/04/2015 | K4290 | Female | 25 | Other | WT | WT | Pan-susceptible |  |  |
|  | 03/27/2018 | KA203 | Male | 50 | Sputum | 35A>G | SIRA | MDR | N | ? |
|  | 08/08/2016 | F187 | Female | 23 | Fiberoptic bronchoscopy lavage fluid | 136delG | SIRECL | XDR |  |  |
|  | 02/12/2018 | K9882 | Male | 75 | Fiberoptic bronchoscopy lavage fluid | 226A>C | SIR | MDR | N | R |
|  | 11/22/2017 | F909 | Male | 60 | Sputum | WT | I | Mono |  |  |
|  | 02/08/2018 | K9859 | Male | 51 | Sputum | 290G>T | SIR | MDR | ND | R |
|  | 03/20/2018 | KA165 | Female | 45 | Sputum | 226A>C | SIR | MDR | N | R |
|  | 11/22/2017 | F906 | Male | 62 | Sputum | WT | WT | Pan-susceptible |  |  |
|  | 01/16/2018 | K9673 | Male | 35 | Sputum | 403_413delACCGATCATTG | SIR | MDR |  |  |
|  | 12/28/2016 | K6818 | Female | 20 | Pyogenic fluid | 226A>C | SR | Other | N | R |
|  | 07/09/2015 | K3462 | Male | 26 | Sputum | 226A>C | SIR | MDR | N | R |
|  | 05/22/2017 | K7793 | Male | 23 | Sputum | WT | WT | Pan-susceptible |  |  |
|  | 01/20/2017 | K6937 | Male | 53 | Fiberoptic bronchoscopy lavage fluid | WT | WT | Pan-susceptible |  |  |
|  | 01/11/2017 | K6907 | Male | 41 | Sputum | WT | R | Mono |  |  |
|  | 11/28/2016 | K6624 | Male | 18 | Ascitic fluid | WT | WT | Pan-susceptible |  |  |
|  | 02/17/2015 | K2642 | Female | 49 | Sputum | 35A>G | SIROAM | XDR | N | ? |
|  | 12/16/2016 | K6736 | Male | 45 | Sputum | 232G>C | IR | MDR |  |  |
|  | 09/09/2017 | K8743 | Female | 51 | Sputum | 407_408insA | SIR | MDR | N | R |
|  | 06/08/2015 | K3282 | Male | 34 | Sputum | WT | O | Mono |  |  |
|  | 08/14/2017 | K8424 | Male | 44 | Sputum | 520delG | SIR | MDR |  |  |
|  | 06/08/2015 | K3251 | Male | 19 | Fiberoptic bronchoscopy lavage fluid | 226A>C | SIROLM | MDR | N | R |
|  | 10/26/2017 | F849 | Female | 41 | Sputum | -11A>C | SIREAL | XDR | N | R |
|  | 10/26/2017 | F843 | Female | 23 | Sputum | 226A>C | SIREL | MDR | N | R |
|  | 11/27/2015 | K4240 | Male | 16 | Sputum | WT | I | Mono |  |  |
|  | 11/02/2017 | F860 | Male | 53 | Sputum | 425C>T | SIREL | MDR | N | R |
|  | 10/13/2017 | F811 | Female | 42 | Sputum | 137C>T | SIREA | MDR | N | R |
|  | 10/26/2017 | F848 | Male | 26 | Sputum | 226A>C | SIREL | MDR | N | R |
|  | 10/26/2017 | F842 | Male | 62 | Sputum | WT | IRE | MDR |  |  |
|  | 11/02/2017 | F865 | Male | 37 | Sputum | 226A>C | SIRE | MDR | N | R |
|  | 10/18/2017 | F826 | Female | 44 | Fiberoptic bronchoscopy lavage fluid | 364C>T | IREL | MDR | N | R |
|  | 10/18/2017 | F838 | Male | 53 | Fiberoptic bronchoscopy lavage fluid | 200C>G | SIREL | MDR |  |  |
|  | 10/26/2017 | F850 | Female | 46 | Sputum | 226A>C | SIREL | MDR | N | R |
|  | 10/26/2017 | F840 | Male | 54 | Other | 226A>C | SIRE | MDR | N | R |
|  | 11/02/2017 | F869 | Female | 17 | Fiberoptic bronchoscopy lavage fluid | 201_202insT | IREL | MDR |  |  |
|  | 11/10/2017 | F879 | Male | 33 | Sputum | 137C>T | IREL | MDR | N | R |
|  | 09/29/2017 | F784 | Female | 34 | Fiberoptic bronchoscopy lavage fluid | 449_450insG | SIRE | MDR | ND | R |
|  | 11/17/2017 | F885 | Female | 54 | Fiberoptic bronchoscopy lavage fluid | 226A>C | SIREL | MDR | N | R |
|  | 11/17/2017 | F884 | Female | 50 | Sputum | 412T>G | IREL | MDR |  |  |
|  | 11/17/2017 | F886 | Female | 17 | Sputum | WT | IRE | MDR |  |  |
|  | 11/17/2017 | F894 | Male | 57 | Pyogenic fluid | 202T>C | SIRE | MDR | N | R |
|  | 11/10/2017 | F870 | Male | 62 | Sputum | 36C>G | SIREA | MDR | N | R |
|  | 11/30/2017 | F921 | Female | 50 | Other | WT | SIR | MDR |  |  |
|  | 11/30/2017 | F918 | Female | 55 | Sputum | WT | IREL | MDR |  |  |
|  | 11/30/2017 | F913 | Male | 45 | Sputum | 188A>G | SIREACL | XDR | N | R |
|  | 11/22/2017 | F902 | Male | 43 | Sputum | 356G>A | SIREL | MDR | ND | R |
|  | 11/22/2017 | F897 | Male | 27 | Sputum | 298A>C | IREAL | XDR | N | R |
|  | 11/17/2017 | F889 | Male | 48 | Sputum | 421C>T | SIREL | MDR | N | R |
|  | 09/29/2017 | F795 | Male | 38 | Other | WT | SIRE | MDR |  |  |
|  | 01/22/2018 | G015 | Male | 28 | Pleural fluid | 403A>C | SIREL | MDR | N | ? |
|  | 01/08/2018 | F957 | Female | 19 | Sputum | 226A>C | SIREL | MDR | N | R |
|  | 06/14/2018 | G413 | Male | 25 | Sputum | WT | SIREL | MDR |  |  |
|  | 01/08/2018 | F965 | Male | 20 | Fiberoptic bronchoscopy lavage fluid | WT | IREL | MDR |  |  |
|  | 01/08/2018 | F966 | Male | 64 | Sputum | 17T>C | SIREAL | XDR | N | R |
|  | 02/07/2017 | F933 | Male | 9 | Sputum | 395G>A | SIREL | MDR | N | R |
|  | 11/22/2017 | F910 | Male | 44 | Sputum | WT | SIRE | MDR |  |  |
|  | 01/18/2018 | F976 | Male | 27 | Pyogenic fluid | 226A>C | SIREL | MDR | N | R |
|  | 01/18/2018 | F981 | Male | 31 | Pyogenic fluid | WT | SIRE | MDR |  |  |
|  | 01/18/2018 | F986 | Male | 53 | Other | 254T>G | SIREAL | XDR | N | R |
|  | 06/28/2018 | G427 | Male | 44 | Fiberoptic bronchoscopy lavage fluid | 425C>G | SIREACL | XDR |  |  |
|  | 06/28/2018 | G422 | Male | 47 | Sputum | WT | SIRE | MDR |  |  |
|  | 02/03/2018 | G046 | Male | 59 | Sputum | 347T>C | SIRE | MDR | ? | R |
|  | 02/03/2018 | G029 | Male | 45 | Sputum | 40T>G | SIREL | MDR | ND | S |
|  | 01/18/2018 | F995 | Male | 34 | Sputum | 347T>C | SIREL | MDR | ? | R |
|  | 01/182018 | F977 | Female | 26 | Sputum | 139A>G | SIRE | MDR | N | ? |
|  | 01/22/2018 | G020 | Male | 61 | Sputum | 416T>G | SIREL | MDR | N | R |
|  | 01/08/2018 | F967 | Male | 23 | Sputum | 254T>G | SIRE | MDR | N | R |
|  | 02/03/2018 | G025 | Female | 51 | Sputum | 500C>T | SIREACL | XDR |  |  |
|  | 02/03/2018 | G026 | Female | 41 | Fiberoptic bronchoscopy lavage fluid | 226A>C | SIRE | MDR | N | R |
|  | 05/31/2018 | G359 | Female | 36 | Sputum | 545T>G | IREL | MDR | ND | R |
|  | 05/31/2018 | G357 | Female | 50 | Fiberoptic bronchoscopy lavage fluid | 29A>C | SIREL | MDR | N | R |
|  | 06/14/2018 | G389 | Female | 20 | Sputum | WT | SIRE | MDR |  |  |
|  | 01/22/2018 | G021 | Male | 20 | Sputum | 226A>C | SIREL | MDR | N | R |
|  | 02/03/2018 | G024 | Male | 54 | Sputum | 529A>C | SIREL | MDR | P | R |
|  | 07/23/2018 | G530 | Male | 34 | Sputum | 289G>T | SIREL | MDR | N | R |
|  | 07/19/2018 | G476 | Female | 36 | Pyogenic fluid | 176C>A | IREL | MDR |  |  |
|  | 06/14/2018 | G395 | Male | 21 | Fiberoptic bronchoscopy lavage fluid | 226A>C | SIRE | MDR | N | R |
|  | 05/31/2018 | G351 | Male | 15 | Sputum | 226A>C | SIRE | MDR | N | R |
|  | 05/31/2018 | G349 | Female | 36 | Sputum | 28C>T | IRE | MDR | N | R |
|  | 07/02/2018 | G459 | Male | 24 | Fiberoptic bronchoscopy lavage fluid | 422A>C | SIREAL | XDR | N | R |
|  | 07/19/2018 | G478 | Male | 44 | Sputum | 40T>G | IR | MDR | ND | S |
|  | 07/19/2018 | G477 | Female | 14 | Sputum | WT | SIR | MDR |  |  |
|  | 05/18/2018 | G325 | Male | 43 | Fiberoptic bronchoscopy lavage fluid | 176C>A | IREL | MDR |  |  |
|  | 07/23/2018 | G535 | Male | 19 | Pyogenic fluid | 394G>A | SIR | MDR | N | R |
|  | 07/02/2018 | G440 | Male | 18 | Sputum | 17T>C | SIREACL | XDR | N | R |
|  | 07/02/2018 | G435 | Male | 37 | Sputum | 40T>C | SIREL | MDR | N | R |
|  | 07/02/2018 | G468 | Male | 64 | Other | 287A>G | SIREAL | XDR | N | R |
|  | 01/22/2018 | G011 | Female | 45 | Pyogenic fluid | 40T>C | SIRE | MDR | N | R |
|  | 01/08/2018 | F962 | Male | 57 | Sputum | 389_390insGG | SIRE | MDR | N | R |
|  | 03/02/2018 | G082 | Male | 28 | Sputum | 524T>C | SIREACL | XDR | N | R |
|  | 03/29/2018 | G173 | Male | 21 | Sputum | 226A>C | SIREL | MDR | N | R |
|  | 08/13/2018 | G607 | Male | 24 | Sputum | 175T>C | SIRE | MDR | N | R |
|  | 08/13/2018 | G610 | Female | 23 | Sputum | 226A>C | SIREL | MDR | N | R |
|  | 05/31/2018 | G345 | Male | 51 | Sputum | WT | SIR | MDR |  |  |
|  | 08/13/2018 | G594 | Male | 45 | Sputum | 422A>C | SIREAL | XDR | N | R |
|  | 08/13/2018 | G615 | Male | 20 | Sputum | 415G>C | SIRECL | XDR | N | R |
|  | 06/14/2018 | G411 | Male | 44 | Sputum | WT | SIR | MDR |  |  |
|  | 05/31/2018 | G343 | Female | 21 | Sputum | 226A>C | SIREL | MDR | N | R |
|  | 06/14/2018 | G410 | Male | 23 | Sputum | 226A>C | SIREL | MDR | N | R |
|  | 06/14/2018 | G384 | Male | 34 | Pleural fluid | WT | SIRE | MDR |  |  |
|  | 04/05/2018 | G210 | Male | 48 | Sputum | -11A>G | SIREA | MDR | N | R |
|  | 04/05/2018 | G207 | Male | 47 | Sputum | 290G>T | SIRE | MDR | ND | R |
|  | 04/19/2018 | G240 | Male | 42 | Sputum | 137_138insC | SIREL | MDR |  |  |
|  | 04/05/2018 | G212 | Male | 32 | Cerebrospinal fluid | 389_390insGG | SIREL | MDR | N | R |
|  | 04/05/2018 | G204 | Male | 25 | Fiberoptic bronchoscopy lavage fluid | 226A>C | SIRE | MDR | N | R |
|  | 06/14/2018 | G402 | Female | 39 | Pyogenic fluid | WT | SIREL | MDR |  |  |
|  | 03/02/2018 | G088 | Male | 46 | Sputum | WT | IR | MDR |  |  |
|  | 02/22/2018 | G064 | Male | 31 | Sputum | 491_492insC | SIREL | MDR |  |  |
|  | 03/15/2018 | G113 | Female | 44 | Sputum | 356G>A | SIREL | MDR | ND | R |
|  | 02/22/2018 | G055 | Female | 17 | Sputum | 347T>G | SIRE | MDR | ND | R |
|  | 03/29/2018 | G168 | Male | 27 | Sputum | WT | IRL | MDR |  |  |
|  | 03/15/2018 | G107 | Male | 56 | Sputum | 35A>C | SIREL | MDR | N | R |
|  | 03/02/2018 | G084 | Male | 53 | Sputum | -11A>G | SIREAL | XDR | N | R |
|  | 02/22/2018 | G062 | Female | 27 | Sputum | 407_408insA | SIREACL | XDR | N | R |
|  | 03/21/2018 | G164 | Male | 69 | Sputum | 226A>C | SIREAL | XDR | N | R |
|  | 03/21/2018 | G162 | Male | 42 | Sputum | 23A>G | SIRL | MDR | N | R |
|  | 03/02/2018 | G091 | Male | 20 | Sputum | 415G>C | SIREAL | XDR | N | R |
|  | 03/21/2018 | G157 | Male | 30 | Sputum | 152A>G | SIRE | MDR | N | R |
|  | 01/22/2018 | G013 | Female | 46 | Sputum | WT | SIRE | MDR |  |  |
|  | 03/29/2018 | G184 | Male | 46 | Sputum | 529A>C | SIRE | MDR | P | R |
|  | 06/14/2018 | G399 | Male | 54 | Sputum | 17T>C | SIREAC | MDR | N | R |
|  | 04/19/2018 | G244 | Female | 27 | Sputum | 20T>G | SIRE | MDR | N | R |
|  | 04/19/2018 | G257 | Female | 31 | Sputum | 29_37delAGAACGACT | IREL | MDR |  |  |
|  | 04/26/2018 | G292 | Male | 45 | Fiberoptic bronchoscopy lavage fluid | 226A>C | SIRE | MDR | N | R |
|  | 04/26/2018 | G297 | Male | 32 | Sputum | 304G>C | SIRE | MDR | ND | S |
|  | 05/18/2018 | G314 | Male | 45 | Sputum | 356G>A | SIRE | MDR | ND | R |
|  | 04/19/2018 | G229 | Female | 63 | Sputum | 538G>T | IRL | MDR | N | R |
|  | 04/05/2018 | G193 | Female | 68 | Fiberoptic bronchoscopy lavage fluid | 40T>C | SIREL | MDR | N | R |
|  | 04/05/2018 | G197 | Male | 46 | Sputum | 226A>C | IRL | MDR | N | R |
|  | 02/03/2018 | G047 | Female | 37 | Sputum | 226A>C | SIREL | MDR | N | R |
|  | 02/03/2018 | G049 | Male | 27 | Sputum | 17T>C | SIREAC | MDR | N | R |
|  | 02/22/2018 | G066 | Female | 23 | Fiberoptic bronchoscopy lavage fluid | 226A>C | SIRE | MDR | N | R |
|  | 02/22/2018 | G068 | Male | 28 | Sputum | WT | SIRE | MDR |  |  |
|  | 05/18/2018 | G334 | Female | 31 | Sputum | 389T>G | SIREACL | XDR | N | R |
|  | 04/19/2018 | G237 | Male | 31 | Sputum | -11A>G | SIRE | MDR | N | R |
|  | 04/19/2018 | G236 | Male | 52 | Sputum | 40T>C | SIRE | MDR | N | R |
|  | 05/18/2018 | G333 | Male | 35 | Sputum | 176C>A | IREL | MDR |  |  |
|  | 05/18/2018 | G321 | Male | 32 | Fiberoptic bronchoscopy lavage fluid | 347T>G | SIRE | MDR | ND | R |
|  | 04/26/2018 | G303 | Male | 18 | Pleural fluid | 226A>C | SIREL | MDR | N | R |
|  | 05/31/2018 | G364 | Male | 38 | Faeces | WT | SIR | MDR |  |  |
|  | 05/18/2018 | G329 | Male | 73 | Sputum | WT | IR | MDR |  |  |
|  | 05/18/2018 | G305 | Female | 25 | Fiberoptic bronchoscopy lavage fluid | 374T>C | IRE | MDR |  |  |
|  | 05/18/2018 | G309 | Female | 33 | Sputum | WT | IRL | MDR |  |  |
|  | 05/18/2018 | G332 | Male | 54 | Sputum | 100T>G | SIREACL | XDR | N | R |
|  | 05/18/2018 | G313 | Female | 15 | Sputum | 356G>A | SIRE | MDR | ND | R |
|  | 03/02/2018 | G081 | Female | 19 | Fiberoptic bronchoscopy lavage fluid | WT | SIRE | MDR |  |  |
|  | 02/22/2018 | G070 | Male | 21 | Fiberoptic bronchoscopy lavage fluid | 422A>C | SIREAL | XDR | N | R |
|  | 05/18/2018 | G310 | Male | 62 | Sputum | 17T>C | SIREACL | XDR | N | R |

S, streptomycin; I, isoniazid; R, rifampicin; E, ethambutol; A, amikacin; C, capreomycin; O, ofloxacin; L, levofloxacin; M, moxifloxacin; N, negative; P, positive; ?, inconsistent results in previously articles; R, resistant; S, sensitive.

Table S2 Mutation types of *pncA* in the 465 clinical isolates

| Mutation types(n=124) | Ratio (%) | Size of mutation |
| --- | --- | --- |
|  |  |  |
| Mutation previously reported(n=94) | 94/124(75.8%) |  |
| Mutation previously not reported(n=30) | 30/124(24.2%) |  |
|  |  |  |
| Single mutation(n=120) | 120/124(96.8%) | 1-21bp |
| Double mutation(n=4) | 4/124(3.2%) | 2bp |
|  |  |  |
| Non-synonymous mutation(n=123) | 123/124(99.2%) | 1-21bp |
| Synonymous mutation(n=1) | 1/124(0.80%) | 1bp |
|  |  |  |
| Nucleotide substitution(n=100) | 100/124(80.6%) |  |
| Amino acid substitution | 90/124(72.6%) | 1-2bp |
| Mutation in upstream region (-11,-12) | 3/124(2.4%) | 1bp |
| Creates stop codon | 7/124(5.6%) | 1bp |
| Nucleotide insertion(n=18) | 18/124(14.5%) | 1-21bp |
| Nucleotide deletion(n=6) | 6/124(4.8%) | 1-11bp |

Table S3 Prediction of PZA susceptibilities of isolates with novel *pncA* mutations using the SUSPECT-PZA webserver

| Strains | Mutations | | PZase  Activity tested by using the *E. coli* ∆*pncA* mutant | Prediction of PZA susceptibility | MIC of PZA  (μg/mL) | No. of  isolates |
| --- | --- | --- | --- | --- | --- | --- |
|  | Nucleotide | Amino acid |  |  |  |  |
| H37Rv | No mutations |  |  |  | 12.5-25 |  |
| 3 | 500C>T | Thr167Ile (T167I) | Positive | Susceptible | ND^b^ | 1 |
| 4 | 274G>C | Ala92Pro (A92P) | Negative | Susceptible | ND | 1 |
| 5 | 374T>C | Val125Ala (V125A) | Positive | Resistant | ND | 1 |
| 6 | 176C>A^a^ and 254T>C | Ser59Tyr (S59Y)  and Leu85Pro (L85P) | Negative | Susceptible and Resistant | >400 | 1 |
| 7 | 403_413delACCGATCATTG | Frameshift | Negative |  | >400 | 2 |
| 8 | 380_381insGG | Frameshift | Negative |  | >400 | 1 |
| 9 | 50_51insC | Frameshift | Negative |  | >400 | 1 |
| 10 | 21_27delCGACGTG | Frameshift | Negative |  | >400 | 1 |
| 11 | 278T>C | Val93Ala (V93A) | Negative | Susceptible | ND | 1 |
| 12 | 29_37delAGAACGACT | Frameshift | Negative |  | ND | 1 |
| 13 | 240_241insT | Frameshift | Negative |  | >400 | 1 |
| 14 | 201_202insT | Frameshift | Negative |  | ND | 1 |
| 15 | 416_417insG | Frameshift | Negative |  | 200 | 3 |
| 16 | 257A>G^a^ and 538G>T | Asp86Gly (D86G)  and Val180Phe (V180F) | Negative | Susceptible and Resistant | 100 | 1 |
| 17 | 412T>G | Cys138Gly(C138G) | Negative | Resistant | ND | 1 |
| 18 | 407_408insTCATTGTGTGCGCCAGACGGC | Frameshift | Negative |  | >400 | 1 |
| 19 | 470_471insGG | Frameshift | Negative |  | ND | 1 |
| 20 | 500_501insCACCGT | Frameshift | Positive |  | <12.5 | 1 |
| 21 | 176C>A | Ser59Tyr(S59Y) | Positive | Susceptible | ND | 3 |
| 22 | 396T>G | Gly132Gly(G132G) | Positive | Susceptible | 25 | 1 |
| 23 | 136delG | Frameshift | Negative |  | >400 | 1 |
| 24 | 520delG | Frameshift | Negative |  | >400 | 2 |
| 25 | 200C>G | Ser67Trp(S67W) | Positive | Susceptible | ND | 1 |
| 26 | 464_465insG | Frameshift | Negative |  | >400 | 1 |
| 27 | 137_138insC | Frameshift | Negative |  | ND | 1 |
| 28 | 491_492insC | Frameshift | Negative |  | ND | 1 |
| 29 | 521_522insT | Frameshift | Negative |  | >400 | 1 |
| 30 | 425C>G | Thr142Arg(T142R) | Negative | Resistant | ND | 1 |
| 31 | 232G>C | Gly78Arg(G78R) | Negative | Resistant | >400 | 1 |
| 32 | 310A>G^a^ and 388G>A^a^ | Ser104Gly(S104G) and Val130Met(V130M) | Negative | Resistant and Resistant | >400 | 1 |

^a^ novel mutation existed in strains with double mutations in *pncA*

^b^ ND, not done

Table S4 Detailed 94 types of previously reported *pncA* mutations identified in this study

| Nucleotide  position and change | Amino-acid  substitution | MIC for PZA(μg/ml)^a^ | PZase activity^a^ | No. of  isolates |
| --- | --- | --- | --- | --- |
| Single mutations |  |  |  |  |
| -12T>C |  | >100 (Mphahlele et al., 2008) | N(Mphahlele et al., 2008) | 1 |
| -11A>C |  | >100 (Jonmalung et al., 2010; Miotto et al., 2014); >900 (Scorpio et al., 1997); >500 (Park et al., 2001) | N (Miotto et al., 2014) | 1 |
| -11A>G |  | >100 (Juréen et al., 2008); >200 (Zhang et al., 2009); >500 (Park et al., 2001);  >900 (Napiórkowska et al., 2014); >25 (Sreevatsan et al., 1997); >300 (Cheng et al., 2000) | N (Park et al., 2001) | 9 |
| 11T>G | Leu4Trp | ND (Lee et al., 2001) | N (Lee et al., 2001) | 1 |
| 17T>C | Ile6Thr | >100 (Juréen et al., 2008); >900 (Bishop et al., 2001) | N (Bishop et al., 2001) | 9 |
| 20T>G | Val7Gly | >100 (Marttila et al., 1999); >800 (Hirano et al., 1997) | N (Akhmetova et al., 2015) | 11 |
| 20T>C | Val7Ala | >100 (Sengstake et al., 2017); 400 (Barco et al., 2006) | N (Barco et al., 2006) | 1 |
| 23A>G | Asp8Gly | >100 (Maslov et al., 2015) | N (Maslov et al., 2015) | 1 |
| 28C>T | Gln10Stop | >100 (Marttila et al., 1999);  >800 (Barco et al., 2006) | N (Barco et al., 2006) | 2 |
| 29A>C | Gln10Pro | >100 (Tan et al., 2014); 800 (Morlock et al., 2000); >900 (Napiórkowska et al., 2014); >800(Hirano et al., 1997) | N (Akhmetova et al., 2015) | 2 |
| 35A>G | Asp12Gly | <100 (Xia et al., 2015); >100 (Mphahlele et al., 2008) | N (Mphahlele et al., 2008) | 5 |
| 35A>C | Asp12Ala | >100 (Cuevas-Cordoba et al., 2013); >200 (Zhang et al., 2009); >900 (Cheng et al., 2000); 1500 (Hou et al., 2000) | N (Lee et al., 2001) | 4 |
| 36C>G | Asp12Glu | >100 (Miotto et al., 2014) | N (Miotto et al., 2014) | 1 |
| 40T>C | Cys14Arg | >100 (Sengstake et al., 2017); >200 (Zhang et al., 2009); >800 (Hirano et al., 1997) | N (Hirano et al., 1997) | 9 |
| 40T>G | Cys14Gly | <100 (Stoffels et al., 2012) | ND (Stoffels et al., 2012) | 2 |
| 41G>A | Cys14Tyr | >500 (Park et al., 2001);>100 (Tracevska et al., 2004) | N (Park et al., 2001) | 1 |
| 56T>G | Leu19Arg | >100 (Jonmalung et al., 2010) | N (Jonmalung et al., 2010) | 1 |
| 71G>A | Gly24Asp | 200 (Ramirez-Busby and Valafar, 2015) | ND(Ramirez-Busby and Valafar, 2015) | 1 |
| 100T>G | Tyr34Asp | >100 (Ando et al., 2010); >400 (Miyagi et al., 2004) | N (Miyagi et al., 2004 | 1 |
| 123C>G | Tyr41Stop | >25 (Sreevatsan et al., 1997); >2000 (Hou et al., 2000) | ND (Hou et al., 2000) | 1 |
| 137C>T | Ala46Val | >100 (Portugal et al., 2004); 400 (Hirano et al., 1997); >900 (Cheng et al., 2000) | N (Portugal et al., 2004) | 2 |
| 139A>G | Thr47Ala | >100 (Cuevas-Cordoba et al., 2013); 12.5 (Morlock et al., 2000); <100 (Somoskovi et al., 2007); >25 (Sreevatsan et al., 1997) | N (Lee et al., 2001) | 2 |
| 143A>C | Lys48Thr | >100 (Ramirez-Busby et al., 2017); <100 (Sheen et al., 2017) | ND (Sheen et al., 2017) | 2 |
| 146A>C | Asp49Ala | >100 (Gu et al., 2016) | N (Lee et al., 2001) | 4 |
| 152A>C | His51Pro | >100 (Portugal et al., 2004); 800 (Morlock et al., 2000);  >200 (Zhang et al., 2009); >800 (Hirano et al., 1997) | N (Hirano et al., 1997) | 1 |
| 152A>G | His51Arg | >100 (Gu et al., 2016); >800 (Barco et al., 2006) | N (Barco et al., 2006) | 1 |
| 171C>G | His57Gln | >100 (Miotto et al., 2014) | N (Miotto et al., 2014) | 1 |
| 174C>A | Phe58Leu | >100 (Chiu et al., 2011) | N (Chiu et al., 2011) | 1 |
| 175T>C | Ser59Pro | >100 (Mphahlele et al., 2008); >800 (Barco et al., 2006) | N(Mphahlele et al., 2008) | 1 |
| 176C>T | Ser59Phe | <100 (Xia et al., 2015) | ND (Xia et al., 2015) | 1 |
| 185C>T | Pro62Leu | >100 (Sheen et al., 2017); >400 (Miyagi et al., 2004 | N (Miyagi et al., 2004 | 9 |
| 187G>C | Asp63His | >500 (Scorpio and Zhang, 1996) | N (Scorpio and Zhang, 1996) | 2 |
| 188A>G | Asp63Gly | >100 (Stoffels et al., 2012); >800 (Hirano et al., 1997);100 (Tracevska et al., 2004) | N (Hirano et al., 1997) | 2 |
| 188A>C | Asp63Ala | >100 (Maslov et al., 2015) | N (Maslov et al., 2015) | 1 |
| 190T>G | Tyr64Asp | >100 (Sheen et al., 2017); >500 (Park et al., 2001) | N (Park et al., 2001) | 2 |
| 202T>C | Trp68Arg | >100 (Miotto et al., 2014);  >200 (Zhang et al., 2009) | N (Miotto et al., 2014) | 2 |
| 203G>T | Trp68Leu | >25 (Sreevatsan et al., 1997); >800 (Barco et al., 2006) | N (Barco et al., 2006) | 1 |
| 204G>A | Trp68Stop | >100 (Miotto et al., 2014) | N (Miotto et al., 2014) | 1 |
| 226A>C | Thr76Pro | >100 (Tan et al., 2014); 400 (Morlock et al., 2000); >200 (Zhang et al., 2009) ; >900 (Napiórkowska et al., 2014); >800 (Hirano et al., 1997); >25 (Sreevatsan et al., 1997) | N (Hirano et al., 1997) | 40 |
| 232G>T | Gly78Cys | >100 (Somoskovi et al., 2007) | ND (Somoskovi et al., 2007) | 1 |
| 232G>A | Gly78Ser | >100 (Ando et al., 2010) | N (Ando et al., 2010) | 1 |
| 241T>G | Phe81Val | >100 (Tan et al., 2014) | ND (Tan et al., 2014) | 1 |
| 254T>G | Leu85Arg | >100 (Tracevska et al., 2004); >400 (Miyagi et al., 2004; >500(Cheng et al., 2000); 1200 (Hou et al., 2000) | N (Lee et al., 2001) | 2 |
| 287A>G | Lys96Arg | >100 (Miotto et al., 2014) | N (Miotto et al., 2014) | 2 |
| 289G>T | Gly97Cys | >100 (Mphahlele et al., 2008) | N (Mphahlele et al., 2008) | 1 |
| 290G>A | Gly97Asp | >100 (Chiu et al., 2011); 400 (Morlock et al., 2000);  >300(Huang et al., 2003); >400 (Miyagi et al., 2004 | N (Chiu et al., 2011) | 1 |
| 290G>T | Gly97Val | >100 (Ramirez-Busby et al., 2017) | ND(Ramirez-Busby et al., 2017) | 2 |
| 291_292insG | Frameshift | 900 (Napiórkowska et al., 2014) | ND(Napiórkowska et al., 2014) | 1 |
| 298A>C | Thr100Pro | >100 (Miotto et al., 2014) | N (Miotto et al., 2014) | 1 |
| 304G>C | Ala102Pro | <100 (Allix-Béguec et al., 2018) | ND(Allix-Béguec et al., 2018) | 1 |
| 308A>G | Tyr103Cys | 900 (Napiórkowska et al., 2014); 1500 (Lemaitre et al., 1999); >100 (Chiu et al., 2011) | N (Chiu et al., 2011) | 1 |
| 309C>G | Tyr103Stop | >100 (Morlock et al., 2000) | N (Morlock et al., 2000) | 1 |
| 322G>C | Gly108Arg | >100(Doustdar et al., 2009);>800 (Barco et al., 2006) | N (Barco et al., 2006) | 3 |
| 329A>G | Asp110Gly | >100 (Xia et al., 2015); 50 (79) | ND (Xia et al., 2015) | 1 |
| 347T>C | Leu116Pro | >100 (Ramirez-Busby and Valafar, 2015) | N&P(Ramirez-Busby and Valafar, 2015) | 4 |
| 347T>G | Leu116Arg | >100 (Marttila et al., 1999); >100 (Somoskovi et al., 2007);>900 (Napiórkowska et al., 2014) | ND (Marttila et al., 1999) | 2 |
| 355T>C | Trp119Arg | >100(Ramirez-Busby and Valafar, 2015) | ND(Ramirez-Busby and Valafar, 2015) | 1 |
| 356G>A | Trp119Stop | >100 (Ramirez-Busby and Valafar, 2015) | ND(Ramirez-Busby and Valafar, 2015) | 4 |
| 359T>G | Leu120Arg | >100(Doustdar et al., 2009); >800(Barco et al., 2006) | N (Barco et al., 2006) | 2 |
| 364C>T | Gln122Stop | >100 (Chiu et al., 2011); >900 (Bishop et al., 2001) | N (Bishop et al., 2001) | 1 |
| 389T>G | Val130Gly | >100(Miotto et al., 2014); >800(Hirano et al., 1997) | N (Hirano et al., 1997) | 3 |
| 389_390insG | Frameshift | >100 (Maslov et al., 2015);  >1600(Aono et al., 2014); >200 (Zhang et al., 2009); >900 (Napiórkowska et al., 2014) | N (Maslov et al., 2015); N&P (Aono et al., 2014) | 1 |
| 389_390insGG | Frameshift | >100 (Portugal et al., 2004); 500 (Lemaitre et al., 1999) | N (Lemaitre et al., 1999) | 8 |
| 394G>A | Gly132Ser | >100 (Chiu et al., 2011); >900  (Scorpio et al., 1997); >500 (Park et al., 2001) | N (Chiu et al., 2011) | 2 |
| 395G>A | Gly132Asp | >100 (Daum et al., 2019); >500 (Park et al., 2001); >2000 (Lemaitre et al., 1999); >800 (Hirano et al., 1997) | N (Hirano et al., 1997) | 32 |
| 395G>T | Gly132Val | >500 (Park et al., 2001); >100 (Doustdar et al., 2009) | N(Park et al., 2001) | 1 |
| 395delG | Frameshift | >100 (Xia et al., 2015) | ND (Xia et al., 2015) | 1 |
| 401C>T | Ala134Val | >100 (Daum et al., 2019); >800 (Morlock et al., 2000); >500 (Park et al., 2001);>300 (Cheng et al., 2000); >400 (Chiu et al., 2011) | N (Cheng et al., 2000) | 1 |
| 403A>C | Thr135Pro | >100 (Miotto et al., 2014);  <100(Stoffels et al., 2012); >800 (Morlock et al., 2000); >500(Park et al., 2001); >900 (Napiórkowska et al., 2014) | N(Park et al., 2001) | 1 |
| 406G>T | Asp136Tyr | >100 (Miotto et al., 2014) | N (Miotto et al., 2014) | 1 |
| 407A>G | Asp136Gly | >500 (Park et al., 2001); >100 (Tan et al., 2014) | N (Park et al., 2001) | 2 |
| 407_408insA | Frameshift | >100 (Miotto et al., 2014) | N (Miotto et al., 2014) | 51 |
| 409C>G | His137Asp | >100 (Miotto et al., 2014) | N (Miotto et al., 2014) | 1 |
| 412T>C | Cys138Arg | >100(Sengstake et al., 2017); >400 (Miyagi et al., 2004 | N (Miyagi et al., 2004 | 1 |
| 415G>A | Val139Met | >100 (Xia et al., 2015); >500 (Cheng et al., 2000) | N (Cheng et al., 2000) | 1 |
| 415G>C | Val139Leu | >100 (Gu et al., 2016); >900(Scorpio et al., 1997); >500 (Park et al., 2001); 900 (Hou et al., 2000) | N (Park et al., 2001) | 2 |
| 416T>G | Val139Gly | >800(Morlock et al., 2000); >100 (Jonmalung et al., 2010) | N (Jonmalung et al., 2010) | 1 |
| 421C>T | Gln141Stop | >100(Maslov et al., 2015); >500 (Park et al., 2001) | N (Park et al., 2001) | 1 |
| 422A>C | Gln141Pro | >100(Marttila et al., 1999); >900 (Scorpio et al., 1997); >200 (Zhang et al., 2009); >300(Cheng et al., 2000); >500 (Scorpio and Zhang, 1996) | N (Scorpio and Zhang, 1996) | 6 |
| 425C>T | Thr142Met | >100 (Jonmalung et al., 2010); >900 (Scorpio et al., 1997);>500 (Park et al., 2001) | N (Jonmalung et al., 2010) | 1 |
| 427G>C | Ala143Pro | >100 (Ramirez-Busby and Valafar, 2015) | ND(Ramirez-Busby and Valafar, 2015) | 1 |
| 436G>A | Ala146Thr | >100 (Jonmalung et al., 2010) | N (Jonmalung et al., 2010) | 1 |
| 449_450insG | Frameshift | >100 (Sengstake et al., 2017) | ND (Sengstake et al., 2017) | 1 |
| 463_464insT | Frameshift | ND (Lee et al., 2001) | N(Lee et al., 2001) | 1 |
| 467T>C | Leu156Pro | >100 (Somoskovi et al., 2007) | ND(Somoskovi et al., 2007) | 2 |
| 515T>G | Leu172Arg | >100 (Stoffels et al., 2012); 300 (Napiórkowska et al., 2014) | ND(Napiórkowska et al., 2014) | 1 |
| 515T>C | Leu172Pro | >100 (Juréen et al., 2008); 200(Morlock et al., 2000); >800 (Hirano et al., 1997) | N (Hirano et al., 1997) | 1 |
| 523A>G | Met175Val | >100 (Stoffels et al., 2012); 300 (Napiórkowska et al., 2014) | N (Miotto et al., 2014) | 2 |
| 524T>C | Met175Thr | >100 (Juréen et al., 2008) | N (Miotto et al., 2014) | 1 |
| 529A>C | Thr177Pro | >100 (Chiu et al., 2011) | P (Chiu et al., 2011) | 2 |
| 538G>T | Val180Phe | >100 (Somoskovi et al., 2007);>200 (Zhang et al., 2009) | N (Zhang et al., 2009) | 1 |
| 539T>C | Val180Ala | >100 (Ramirez-Busby and Valafar, 2015) | ND(Ramirez-Busby and Valafar, 2015) | 1 |
| 545T>G | Leu182Trp | >100 (Sheen et al., 2017) | ND (Sheen et al., 2017) | 2 |
| Double mutations |  |  |  |  |
| 193T>C; 359T>C | Ser65Pro; Leu120Pro | 193T>C,ND (Ramirez-Busby and Valafar, 2015);  359T>C, >100 (Portugal et al., 2004) | 193T>C, P (Ramirez-Busby and Valafar, 2015);  359T>C, N (Portugal et al., 2004) | 1 |

^a^ If previously reported, the values of MIC and PZase activity were quoted from the references.

N, negative; P, positive; ND, not determined

**References**

Akhmetova, A., Kozhamkulov, U., Bismilda, V., Chingissova, L., Abildaev, T., Dymova, M., et al. (2015). Mutations in the pncA and rpsA genes among 77 Mycobacterium tuberculosis isolates in Kazakhstan. *Int J Tuberc Lung Dis* 19(2)**,** 179-184. doi: 10.5588/ijtld.14.0305.

Allix-Béguec, C., Arandjelovic, I., Bi, L., Beckert, P., Bonnet, M., Bradley, P., et al. (2018). Prediction of Susceptibility to First-Line Tuberculosis Drugs by DNA Sequencing. *N Engl J Med* 379(15)**,** 1403-1415. doi: 10.1056/NEJMoa1800474.

Ando, H., Mitarai, S., Kondo, Y., Suetake, T., Sekiguchi, J.I., Kato, S., et al. (2010). Pyrazinamide resistance in multidrug-resistant Mycobacterium tuberculosis isolates in Japan. *Clin Microbiol Infect* 16(8)**,** 1164-1168. doi: 10.1111/j.1469-0691.2009.03078.x.

Aono, A., Chikamatsu, K., Yamada, H., Kato, T., and Mitarai, S. (2014). Association between pncA gene mutations, pyrazinamidase activity, and pyrazinamide susceptibility testing in Mycobacterium tuberculosis. *Antimicrob Agents Chemother* 58(8)**,** 4928-4930. doi: 10.1128/aac.02394-14.

Barco, P., Cardoso, R.F., Hirata, R.D., Leite, C.Q., Pandolfi, J.R., Sato, D.N., et al. (2006). pncA mutations in pyrazinamide-resistant Mycobacterium tuberculosis clinical isolates from the southeast region of Brazil. *J Antimicrob Chemother* 58(5)**,** 930-935. doi: 10.1093/jac/dkl363.

Bishop, K.S., Blumberg, L., Trollip, A.P., Smith, A.N., Roux, L., York, D.F., et al. (2001). Characterisation of the pncA gene in Mycobacterium tuberculosis isolates from Gauteng, South Africa. *Int J Tuberc Lung Dis* 5(10)**,** 952-957.

Cheng, S.J., Thibert, L., Sanchez, T., Heifets, L., and Zhang, Y. (2000). pncA mutations as a major mechanism of pyrazinamide resistance in Mycobacterium tuberculosis: spread of a monoresistant strain in Quebec, Canada. *Antimicrob Agents Chemother* 44(3)**,** 528-532.

Chiu, Y.C., Huang, S.F., Yu, K.W., Lee, Y.C., Feng, J.Y., and Su, W.J. (2011). Characteristics of pncA mutations in multidrug-resistant tuberculosis in Taiwan. *BMC Infect Dis* 11**,** 240. doi: 10.1186/1471-2334-11-240.

Cuevas-Cordoba, B., Xochihua-Gonzalez, S.O., Cuellar, A., Fuentes-Dominguez, J., and Zenteno-Cuevas, R. (2013). Characterization of pncA gene mutations in pyrazinamide-resistant Mycobacterium tuberculosis isolates from Mexico. *Infect Genet Evol* 19**,** 330-334. doi: 10.1016/j.meegid.2012.12.013.

Daum, L.T., Konstantynovska, O.S., Solodiankin, O.S., Poteiko, P.I., Bolotin, V.I., Rodriguez, J.D., et al. (2019). Characterization of novel Mycobacterium tuberculosis pncA gene mutations in clinical isolates from the Ukraine. *Diagn Microbiol Infect Dis* 93(4)**,** 334-338. doi: 10.1016/j.diagmicrobio.2018.10.018.

Doustdar, F., Khosravi, A.D., and Farnia, P. (2009). Mycobacterium tuberculosis genotypic diversity in pyrazinamide-resistant isolates of Iran. *Microb Drug Resist* 15(4)**,** 251-256. doi: 10.1089/mdr.2009.0066.

Gu, Y., Yu, X., Jiang, G., Wang, X., Ma, Y., Li, Y., et al. (2016). Pyrazinamide resistance among multidrug-resistant tuberculosis clinical isolates in a national referral center of China and its correlations with pncA, rpsA, and panD gene mutations. *Diagn Microbiol Infect Dis* 84(3)**,** 207-211. doi: 10.1016/j.diagmicrobio.2015.10.017.

Hirano, K., Takahashi, M., Kazumi, Y., Fukasawa, Y., and Abe, C. (1997). Mutation in pncA is a major mechanism of pyrazinamide resistance in Mycobacterium tuberculosis. *Tuber Lung Dis* 78(2)**,** 117-122.

Hou, L., Osei-Hyiaman, D., Zhang, Z., Wang, B., Yang, A., and Kano, K. (2000). Molecular characterization of pncA gene mutations in Mycobacterium tuberculosis clinical isolates from China. *Epidemiol Infect* 124(2)**,** 227-232. doi: 10.1017/s0950268899003635.

Huang, T.S., Lee, S.S., Tu, H.Z., Huang, W.K., Chen, Y.S., Huang, C.K., et al. (2003). Correlation between pyrazinamide activity and pncA mutations in Mycobacterium tuberculosis isolates in Taiwan. *Antimicrob Agents Chemother* 47(11)**,** 3672-3673. doi: 10.1128/aac.47.11.3672-3673.2003.

Jonmalung, J., Prammananan, T., Leechawengwongs, M., and Chaiprasert, A. (2010). Surveillance of pyrazinamide susceptibility among multidrug-resistant Mycobacterium tuberculosis isolates from Siriraj Hospital, Thailand. *BMC Microbiol* 10**,** 223. doi: 10.1186/1471-2180-10-223.

Juréen, P., Werngren, J., Toro, J.C., and Hoffner, S. (2008). Pyrazinamide resistance and pncA gene mutations in Mycobacterium tuberculosis. *Antimicrob Agents Chemother* 52(5)**,** 1852-1854. doi: 10.1128/aac.00110-08.

Lee, K.W., Lee, J.M., and Jung, K.S. (2001). Characterization of pncA mutations of pyrazinamide-resistant Mycobacterium tuberculosis in Korea. *J Korean Med Sci* 16(5)**,** 537-543. doi: 200110537 [pii].

Lemaitre, N., Sougakoff, W., Truffot-Pernot, C., and Jarlier, V. (1999). Characterization of new mutations in pyrazinamide-resistant strains of Mycobacterium tuberculosis and identification of conserved regions important for the catalytic activity of the pyrazinamidase PncA. *Antimicrob Agents Chemother* 43(7)**,** 1761-1763.

Marttila, H.J., Marjamäki, M., Vyshnevskaya, E., Vyshnevskiy, B.I., Otten, T.F., Vasilyef, A.V., et al. (1999). pncA mutations in pyrazinamide-resistant Mycobacterium tuberculosis isolates from northwestern Russia. *Antimicrob Agents Chemother* 43(7)**,** 1764-1766.

Maslov, D.A., Zaĭchikova, M.V., Chernousova, L.N., Shur, K.V., Bekker, O.B., Smirnova, T.G., et al. (2015). Resistance to pyrazinamide in Russian Mycobacterium tuberculosis isolates: pncA sequencing versus Bactec MGIT 960. *Tuberculosis (Edinb)* 95(5)**,** 608-612. doi: 10.1016/j.tube.2015.05.013.

Miotto, P., Cabibbe, A.M., Feuerriegel, S., Casali, N., Drobniewski, F., Rodionova, Y., et al. (2014). Mycobacterium tuberculosis pyrazinamide resistance determinants: a multicenter study. *mBio* 5(5)**,** e01819-01814. doi: 10.1128/mBio.01819-14.

Miyagi, C., Yamane, N., Yogesh, B., Ano, H., and Takashima, T. (2004). Genetic and phenotypic characterization of pyrazinamide-resistant mycobacterium tuberculosis complex isolates in Japan. *Diagn Microbiol Infect Dis* 48(2)**,** 111-116. doi: 10.1016/j.diagmicrobio.2003.09.013.

Morlock, G.P., Crawford, J.T., Butler, W.R., Brim, S.E., Sikes, D., Mazurek, G.H., et al. (2000). Phenotypic characterization of pncA mutants of Mycobacterium tuberculosis. *Antimicrob Agents Chemother* 44(9)**,** 2291-2295. doi: 10.1128/aac.44.9.2291-2295.2000.

Mphahlele, M., Syre, H., Valvatne, H., Stavrum, R., Mannsåker, T., Muthivhi, T., et al. (2008). Pyrazinamide resistance among South African multidrug-resistant Mycobacterium tuberculosis isolates. *J Clin Microbiol* 46(10)**,** 3459-3464. doi: 10.1128/jcm.00973-08.

Napiórkowska, A., Rüsch-Gerdes, S., Hillemann, D., Richter, E., and Augustynowicz-Kopeć, E. (2014). Characterisation of pyrazinamide-resistant Mycobacterium tuberculosis strains isolated in Poland and Germany. *Int J Tuberc Lung Dis* 18(4)**,** 454-460. doi: 10.5588/ijtld.13.0457.

Park, S.K., Lee, J.Y., Chang, C.L., Lee, M.K., Son, H.C., Kim, C.M., et al. (2001). pncA mutations in clinical Mycobacterium tuberculosis isolates from Korea. *BMC Infect Dis* 1**,** 4. doi: 10.1186/1471-2334-1-4.

Portugal, I., Barreiro, L., Moniz-Pereira, J., and Brum, L. (2004). pncA mutations in pyrazinamide-resistant Mycobacterium tuberculosis isolates in Portugal. *Antimicrob Agents Chemother* 48(7)**,** 2736-2738. doi: 10.1128/aac.48.7.2736-2738.2004.

Ramirez-Busby, S.M., and Valafar, F. (2015). Systematic review of mutations in pyrazinamidase associated with pyrazinamide resistance in Mycobacterium tuberculosis clinical isolates. *Antimicrob Agents Chemother* 59(9)**,** 5267-5277. doi: 10.1128/aac.00204-15.

Ramirez-Busby, S.M., Rodwell, T.C., Fink, L., and Catanzaro, D. (2017). A Multinational Analysis of Mutations and Heterogeneity in PZase, RpsA, and PanD Associated with Pyrazinamide Resistance in M/XDR Mycobacterium tuberculosis. 7(1)**,** 3790. doi: 10.1038/s41598-017-03452-y.

Scorpio, A., Lindholm-Levy, P., Heifets, L., Gilman, R., Siddiqi, S., Cynamon, M., et al. (1997). Characterization of pncA mutations in pyrazinamide-resistant Mycobacterium tuberculosis. *Antimicrob Agents Chemother* 41(3)**,** 540-543.

Scorpio, A., and Zhang, Y. (1996). Mutations in pncA, a gene encoding pyrazinamidase/nicotinamidase, cause resistance to the antituberculous drug pyrazinamide in tubercle bacillus. *Nature medicine* 2(6)**,** 662-667.

Sengstake, S., Bergval, I.L., Schuitema, A.R., de Beer, J.L., Phelan, J., de Zwaan, R., et al. (2017). Pyrazinamide resistance-conferring mutations in pncA and the transmission of multidrug resistant TB in Georgia. *BMC Infect Dis* 17(1)**,** 491. doi: 10.1186/s12879-017-2594-3.

Sheen, P., Requena, D., Gushiken, E., Gilman, R.H., Antiparra, R., Lucero, B., et al. (2017). A multiple genome analysis of Mycobacterium tuberculosis reveals specific novel genes and mutations associated with pyrazinamide resistance. 18(1)**,** 769. doi: 10.1186/s12864-017-4146-z.

Somoskovi, A., Dormandy, J., Parsons, L.M., Kaswa, M., Goh, K.S., Rastogi, N., et al. (2007). Sequencing of the pncA gene in members of the Mycobacterium tuberculosis complex has important diagnostic applications: Identification of a species-specific pncA mutation in "Mycobacterium canettii" and the reliable and rapid predictor of pyrazinamide resistance. *J Clin Microbiol* 45(2)**,** 595-599. doi: 10.1128/jcm.01454-06.

Sreevatsan, S., Pan, X., Zhang, Y., Kreiswirth, B., and Musser, J. (1997). Mutations associated with pyrazinamide resistance in pncA of Mycobacterium tuberculosis complex organisms. *Antimicrobial agents and chemotherapy* 41(3)**,** 636.

Stoffels, K., Mathys, V., Fauville-Dufaux, M., Wintjens, R., and Bifani, P. (2012). Systematic analysis of pyrazinamide-resistant spontaneous mutants and clinical isolates of Mycobacterium tuberculosis. *Antimicrob Agents Chemother* 56(10)**,** 5186-5193. doi: 10.1128/aac.05385-11.

Tan, Y., Hu, Z., Zhang, T., Cai, X., Kuang, H., Liu, Y., et al. (2014). Role of pncA and rpsA gene sequencing in detection of pyrazinamide resistance in Mycobacterium tuberculosis isolates from southern China. *J Clin Microbiol* 52(1)**,** 291-297. doi: 10.1128/jcm.01903-13.

Tracevska, T., Jansone, I., Baumanis, V., Nodieva, A., Marga, O., and Skenders, G. (2004). Spectrum of pncA mutations in multidrug-resistant Mycobacterium tuberculosis isolates obtained in Latvia. *Antimicrob Agents Chemother* 48(8)**,** 3209-3210. doi: 10.1128/aac.48.8.3209-3210.2004.

Xia, Q., Zhao, L.L., Li, F., Fan, Y.M., Chen, Y.Y., Wu, B.B., et al. (2015). Phenotypic and genotypic characterization of pyrazinamide resistance among multidrug-resistant Mycobacterium tuberculosis isolates in Zhejiang, China. *Antimicrob Agents Chemother* 59(3)**,** 1690-1695. doi: 10.1128/aac.04541-14.

Zhang, H., Bi, L.J., Li, C.Y., Sun, Z.G., Deng, J.Y., and Zhang, X.E. (2009). Mutations found in the pncA gene of Mycobacterium tuberculosis in clinical pyrazinamide-resistant isolates from a local region of China. *J Int Med Res* 37(5)**,** 1430-1435. doi: 10.1177/147323000903700517.
